# Inhibiting YAP in Endothelial Cells From Entering the Nucleus Attenuates Blood-Brain Barrier Damage During Ischemia-Reperfusion Injury

**DOI:** 10.3389/fphar.2021.777680

**Published:** 2021-11-26

**Authors:** Shuaishuai Gong, Huifen Ma, Fan Zheng, Juan Huang, Yuanyuan Zhang, Boyang Yu, Fang Li, Junping Kou

**Affiliations:** State Key Laboratory of Natural Medicines, Jiangsu Key Laboratory of TCM Evaluation and Translational Research, Department of Pharmacology of Chinese Material Medical, School of Traditional Pharmacy, China Pharmaceutical University, Nanjing, China

**Keywords:** YAP, verteporfin, endothelial cells, blood-brain barrier, ischemic stroke

## Abstract

Blood-brain barrier (BBB) damage is a critical event in ischemic stroke, contributing to aggravated brain damage. Endothelial cell form a major component of the BBB, but its regulation in stroke has yet to be clarified. We investigated the function of Yes-associated protein 1 (YAP) in the endothelium on BBB breakdown during cerebral ischemia/reperfusion (I/R) injury. The effects of YAP on BBB dysfunction were explored in middle cerebral artery occlusion/reperfusion (MCAO/R)-injury model mice and using brain microvascular endothelial cells (BMEC) exposed to oxygen-glucose deprivation/reoxygenation (OGD/R) injury. The degree of brain injury was estimated using staining (2,3,5-Triphenyltetrazolium chloride, hematoxylin and eosin) and the detection of cerebral blood flow. BBB breakdown was investigated by examining the leakage of Evans Blue dye and evaluating the expression of tight junction (TJ)-associated proteins and matrix metallopeptidase (MMP) 2 and 9. YAP expression was up-regulated in the nucleus of BMEC after cerebral I/R injury. Verteporfin (YAP inhibitor) down-regulated YAP expression in the nucleus and improved BBB hyperpermeability and TJ integrity disruption stimulated by cerebral I/R. YAP-targeted small interfering RNA (siRNA) exerted the same effects in BMEC cells exposed to OGD/R injury. Our findings provide new insights into the contributions made by YAP to the maintenance of BBB integrity and highlight the potential for YAP to serve as a therapeutic target to modulate BBB integrity following ischemic stroke and related cerebrovascular diseases.

## 1 Introduction

Ischemic stroke is often accompanied by vascular dysfunction due to damage to the blood-brain barrier (BBB) (Feigin et al., 2018; Ozen et al., 2018). The BBB is a specialized barrier comprised of endothelial cells (ECs), tight junctions (TJs), pericytes, astrocytic end-feet processes, and the basement membrane. These components are crucial for the establishment of a highly regulated microenvironment, which ensures appropriate neuronal function (Moskowitz et al., 2010; Lallukka et al., 2018; Sweeney et al., 2019). Therefore, protection against BBB destruction represents an effective strategy for the clinical prevention and treatment of ischemic stroke.

ECs represent the most important component of the BBB, lining the entire microvasculature, and forming TJs to limit paracellular transport, ECs also display considerably a limited rate of transcellular transport for hydrophilic molecules, which contributes to the maintenance of barrier function (Armulik et al., 2010; Guclu et al., 2014). The appropriate regulation and maintenance of the barrier integrity of the ECs that line within blood vessels represent an essential feature of the BBB. The prevention of early cytoskeletal changes in microvascular ECs can attenuate BBB breakdown and secondary tissue injury, resulting in the amelioration of long-term neurological deficits (Fernandez-Klett et al., 2013; Hall et al., 2014). However, the molecular mechanisms that underlie the regulation of EC function and the associated BBB alterations that occur under pathological conditions remain incompletely understood.

The Hippo/Yes-associated protein 1 (YAP) kinase cascade has been reported to serve as a critical regulator of organ size, tissue regeneration, and tumor suppression (Halder and Johnson, 2011; Lin et al., 2017). The Hippo pathway negatively regulates the activity of transcriptional co-activators, including YAP and transcriptional co-activator with PDZ-binding motif (TAZ) (Varelas, 2014). In the nucleus, YAP transcribes genes that control cell proliferation, apoptosis, and cell fate (Szymaniak et al., 2015). YAP localization becomes dramatically altered upon tissue damage, and in some tissues, nuclear YAP abundance is associated with increased regeneration (Choi and Kwon, 2015). YAP has been shown to be involved in BBB dysfunction during ischemic stroke (Ouyang et al., 2020; Gong et al., 2019), although the function of YAP in the maintenance of the cerebral endothelial barrier (CEB) remains unclear. We postulated that YAP might be essential for EC protection and the maintenance of CEB integrity following ischemic stroke.

In the present study, we investigated the effects of YAP on the CEB in mouse and cell models of ischemic-reperfusion injury, with the aim of determining whether YAP represents a potential therapeutic target for regulating BBB integrity after ischemic stroke and related cerebrovascular diseases.

## 2 Materials and Methods

### 2.1 Ethical Approval of the Study Protocol

The welfare of all animals was ensured, and all experimental procedures were performed in accordance with the Guide for the Care and Use of Laboratory Animals established by the National Institutes of Health. The Animal Ethics Committee of China Pharmaceutical University (Nanjing, China) approved all protocols [No. SYXK(Su)2018-0008].

### 2.2 Animals

Male C57BL/6J mice were purchased from the Animal Center of Yangzhou University (Yangzhou, China). Adequate food and water were provided. Animals were housed in cage in an environment maintained at a constant temperature (22–24°C) with a normal circadian rhythm.

### 2.3 Cell Culture

bEnd.3 cells were purchased from Bioleaf Biotech (Shanghai, P.R. China) and cultured in Dulbecco’s modified Eagle’s medium (DMEM; Gibco, Billings, MT, United States) supplemented with 15% fetal bovine serum (FBS; Gibco), 100 U/mL penicillin and 100 U/mL streptomycin (Ameresco, Framingham, MA, United States) at 37°C in a humidified atmosphere of 5% CO_2_ and 95% air. Cells were plated onto cell culture dishes and grown to 80–90% confluence before experimentations.

### 2.4 Middle Cerebral Artery Occlusion/Reperfusion Model

Mice were anesthetized in an induction chamber using 3–4% isoflurane in 30% O_2_/70% N_2_. Anesthetization was confirmed after approximately 2 min when respiration slowed to one breathe per second. Animals were removed from the induction chamber and placed in an anesthesia mask, which maintained an isoflurane concentration of 1–1.5%. Middle cerebral artery occlusion/reperfusion (MCAO/R) was induced using a method based on intraluminal filaments with slight modification as described previously (Gong et al., 2019). Briefly, the right middle cerebral artery of mouse was occluded by inserting a blunt-tip 4-0 nylon monofilament for 1 h followed with reperfusion for 24 h.

### 2.5 Oxygen and Glucose Deprivation/Reperfusion Model

bEnd.3 cells were placed in a 37°C anaerobic chamber (0.2% O_2_, 5% CO_2_, 95% N_2_) and cultured in glucose-free medium for 6 h. After the oxygen-glucose deprivation, the cells were placed in glucose-containing DMEM with 15% FBS and incubated under normoxic conditions for hours in order to imitate I/R-like conditions Cao et al. (2016).

### 2.6 Transendothelial Electrical Resistance Assay

The protective effects of verteporfin (VP, CAS No. 129497-78-5) were examined *in vitro*. Cells were divided into four groups (*n* = 3): Control, OGD/R, VP (1 μM, Supplementary Figure S1) treatment after 6 h OGD, and edaravone (Edara, 1 μM, CAS No. 89-25-8). Edara is a commonly used drug for the clinical treatment of ischemic stroke and is often used as a positive drug in basic research (Bao et al., 2018). bEnd.3 cells were cultured on top of gelatin-coated transwell inserts in 24-well plates for 7 days. The Transendothelial electrical resistance (TEER) of the EC monolayer was monitored daily using a Millicell-ERS voltohmmeter (Millipore, United States). The results obtained from the experimental groups were measured after subtracting the value of a blank, cell-free filter.

### 2.7 TTC Staining

The protective effects of VP were examined in mice that were randomly divided into four groups (*n* = 6 per group): sham, MCAO/R, VP (10 mg/kg, i. p.) after 1 h MCAO, and Edara (5 mg/kg, i. p.). After 24 h of reperfusion, the mice brains were quickly removed and frozen at −70°C. The frozen brain was coronally cut into five slices, then incubated individually using a 24-well culture plate with 1% triphenyl tetrazolium chloride (TTC) solution at 37°C for 15 min. The infarct area was measured by computerized planimetry after photographing with a digital camera. The infarct volume is calculated by summing infarct areas on each slice and multiplying by slice thickness. The personnel conducting the TTC staining was blinded to the study group assignment in order to avoid subjective factors affecting the experimental results Tsubokawa et al. (2017).

### 2.8 Determination of Cerebral Edema and Neurological Deficits

The mice were sacrificed after MCAO/R induction. The brains were taken out and the wet weight of tissue was accurately measured. After dried in an oven at 100°C for 48 h, the lung tissues were weighed again, recording as dry weight. The content of water in brain were calculated to determine the degree of brain edema. The neurological deficits of the experimental animals were graded on an 18-point scale, as previously described Wu et al. (2019). The evaluation indicators include body symmetry, gait, climbing, circling behavior, forelimb symmetry, compulsory circling and whisker response. The index scores are added together as the final score. The higher the score, the more severe the neurological deficit.

### 2.9 Hematoxylin and Eosin Staining

Animal brains were removed 24 h after reperfusion, mice were euthanized, the brains excised rapidly, and dipped in 4% paraformaldehyde. Examination was completed in the Pathology Department of the Jiangsu Center for Safety Evaluation of Drugs (Jiangsu, P.R. China) and the brain slices were observed by a digital scanner (NanoZoomer 2.0 RS, Hamamatsu, Japan).

### 2.10 Cerebral Blood Flow Measurement

After anesthesia with 3% pentobarbital sodium, an incision of about 1–2 cm was made in the abdominal cavity of mice. Cerebral blood flow (CBF) in the mesentery was measured using a laser Doppler flowmeter Laser. Images were acquired at ischemia onset and during reperfusion (*n* = 6 per group).

### 2.11 Evans Blue Analysis

Evans Blue (EB) extravasation was used to determine BBB integrity as described previously Wu et al. (2019). At 22 h after reperfusion, 2% EB dye (3 ml/kg, Sigma, United States) was injected *via* the tail vein. The mice were euthanized at 2 h after injection of EB and then perfused with saline. The brains were rapidly taken out and imaged. Then the right hemisphere of brain tissue was weighed, homogenized in formamide (0.1 g/ml) and centrifuged at 5,000 g for 30 min after incubated at 60°C for 18 h. The supernatants were collected to determine the quantity of EB, the absorbance at 620 nm was measured spectrophotometrically using an Infinite M200 Pro plate reader (Tecan, NC, United States). EB leakage into the brain tissue was assessed with a standard curve and expressed as micrograms per Gram of wet brain tissue.

### 2.12 *In vitro* Permeability Assay

Cells were incubated in the Millicell™ cell culture inserts in a humidified atmosphere of 5% CO_2_ and 95% air for 7 days. After exposure to OGD/R conditions and drugs, the medium was removed. 200 μL of EB solution (0.67 mg evans blue powder dissolved in 4% BSA solution) were added into the Millicell cell culture inserts and 600 μL of 4% BSA solution were added into the external chamber. The cells were continuously incubated for another 1 h and then the external solution was collected and the absorbance at 620 nm was measured spectrophotometrically using an Infinite M200 Pro plate reader (Tecan, NC, United States). The EB leakage of each group was calculated according to the standard curve and expressed as a percentage of the values of control group.

### 2.13 siRNA Transfection and Plasmid

#### 2.13.1 YAP-siRNA Treatment

YAP-siRNA (sense: 5′-GAC​AUC​UUC​UGG​UCA​GAG​A-3′, and anti-sense: 3′-AGU​ACC​GGA​GGU​AAC​AGA​G-5′) were constructed by Genomeditech Co., Ltd, (Shanghai, China). Cells were divided randomly into four groups: Control, Control + siRNA, OGD/R, OGD/R + siRNA. bEnd.3 cells were treated with YAP-siRNA or control solvent for 6 h in DEME medium and proliferated for another 24 h. OGD/R was treated subsequently in DMEM medium. After testing the expression of YAP by western blot, the cells with adequate interference efficiency were used in the evaluation of the downstream signaling pathways.

### 2.14 Cell Viability

Culture medium containing 5 mg/ml 3-(4,5-Dimethylthiazol-2-yl)-2,5-diphenyltetrazolium bromide (MTT) solution replaced the complete medium Four hours after incubation at 37°C, the reaction solution was removed, and 150 µL DMSO was added to each well. A microplate reader (Epoch, Bio Tek, Winooski, VT, United States) was used to record the absorbance with dual waves at 570 and 650 nm after 10 min of shaking.

### 2.15 Western Blot Analysis

The cells or brain tissue samples (*n* = 6, for each group) were decapitated and rapidly collected. The prepared cells or tissues (brain tissues from the ischemic penumbra) were homogenized in 1:10 (w/v) ice-cold protein extraction buffer in glass homogenizers. To detect the levels of YAP and phospho- (p)-YAP in the nucleus, a Nuclear Extraction Kit (Solarbio, Cat: SN0020) was used to isolate and purify nuclear and cytoplasmic fractions. To examine the levels of zonula occludens-1 (ZO-1), occludin, matrix metalloproteinase (MMP)-2 and MMP-9, soluble proteins were extracted from cell lysates by centrifugation at 12,000 × *g* for 10 min at 4°C and collecting the supernatant. The membranes were blocked with phosphate-buffered saline containing Tween20 (PBST) containing 5% skim milk for 2 h at room temperature and then incubated with primary rabbit monoclonal antibody overnight at 4°C (YAP, p-YAP, 1:500; Proteintech Group, United States ZO-1, occludin 1:500; Abcam, United Kingdom MMP-2, MMP-9, 1:800; CST, United States). The membranes were then washed and incubated with secondary antibody (anti-rabbit IgG, 1:3,000; Proteintech Group, United States) for 1.5 h at room temperature. The anti-actin antibody (1:1,000; Proteintech Group, United States) served as a loading control. The protein bands were visualized with enhanced chemiluminescence reagents (ECL), and the signal densitometry was quantified using a western blotting detection system (Quantity One, Bio-Rad Laboratories, United States) by an observer blinded to the groups of animals or cells being examined.

### 2.16 *In vivo* and *In vitro* Immunofluorescence

Specimens were sectioned at thickness 10 µm to adhesive slides and bEnd.3 cells were cultured on laser confocal dishes. Specimens were treated with blocking buffer (5% bovine serum albumin, 0.2%Triton-100) for 1 h at 4 °C and then incubated overnight at 4°C with primary antibody against YAP, p-YAP, and ZO-1 (ZO-1, 1:200; Proteintech Group, United States; YAP, 1:300; p-YAP, 1:100; Abcam, United Kingdom), followed by incubation with an Alexa Fluor 488-conjugated donkey anti-rabbit IgG (HþL) antibody (Invitrogen, Carlsbad, CA, United States) and 4′,6-Diamidino-2-phenylindole (Beyotime Biotechnology). Fluorescent images were observed with a confocal laser scanning microscope (LSM700; Zeiss, Jena, Germany) and processed using ZEN imaging software. Regarding the IF brain slice, the location of the studied brain area was showed as an illustration figure in Supplementary Figure S1.

### 2.17 Statistical Analysis

Data are expressed as the mean ± SEM. Statistical analyses were carried out using the Student’s t-test (two-tailed) for comparison between two groups and one-way analysis of variance (ANOVA) followed by Dunnett’s test if the data involved three or more groups. Tests were considered significant at *p* < 0.05. Analyses were carried out using Prism v5.01 (GraphPad, San Diego, CA, United States).

## 3 Results

YAP is highly expressed in the nuclei of brain endothelial cells from mice subjected to MCAO/R injury *in vivo*.

**FIGURE 1:**
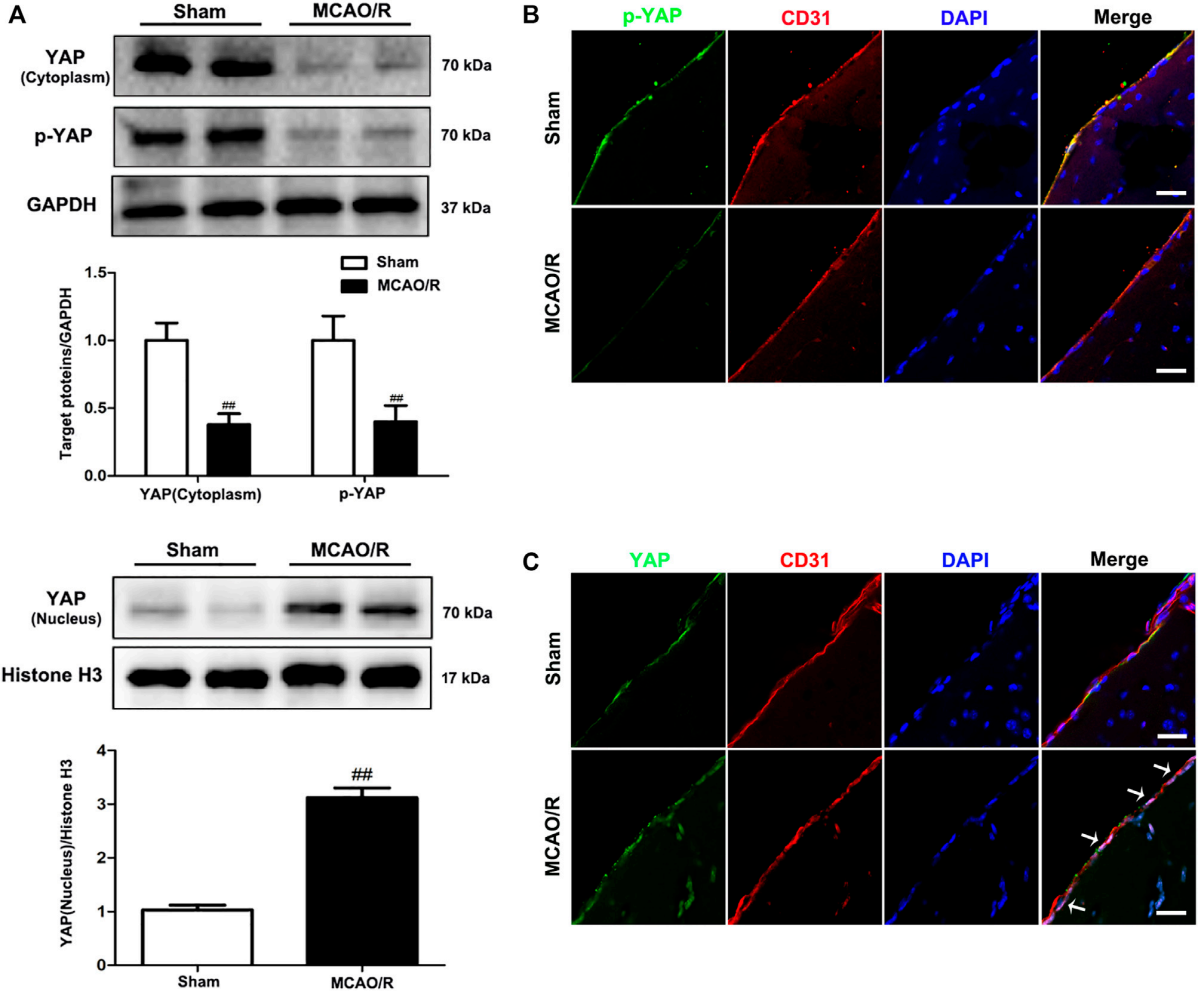
Expression of YAP/p-YAP in the brain endothelial cells following MCAO/R-induced injury. Mice were subjected to 1 h of ischemia and 24 h of reperfusion. **(A)** MCAO/R-induced changes in YAP/p-YAP protein expression levels in the cytoplasm and nucleus were detected by western blotting analysis. **(B)** and **(C)** MCAO/R-induced changes in YAP/p-YAP protein expression levels were detected by immunofluorescence analysis using a combination of anti-YAP (green), anti-p-YAP (green), CD31 (red) and DAPI (blue) staining in mice. The white arrow represents the number of YAP protein into the nucleus. Scale bar = 50 µm. Data are expressed as the mean ± SD, *n* = 3. ^##^ *p* < 0.01 *vs*. Sham group.

To determine the specific role played by YAP in ischemic stroke, YAP expression levels were evaluated in the brain after MCAO/R injury using western blotting and IF analyses. After 1 h of cerebral ischemia and 24 h of reperfusion, the expression levels of YAP and p-YAP were reduced in the cytoplasm, and the expression level of YAP was significantly increased in the nucleus (Figure 1 and Supplementary Figures S3–5).

Verteporfin (a small molecule inhibitor of YAP) inhibits the expression of YAP in the nucleus under MCAO/R injury conditions.

**FIGURE 2:**
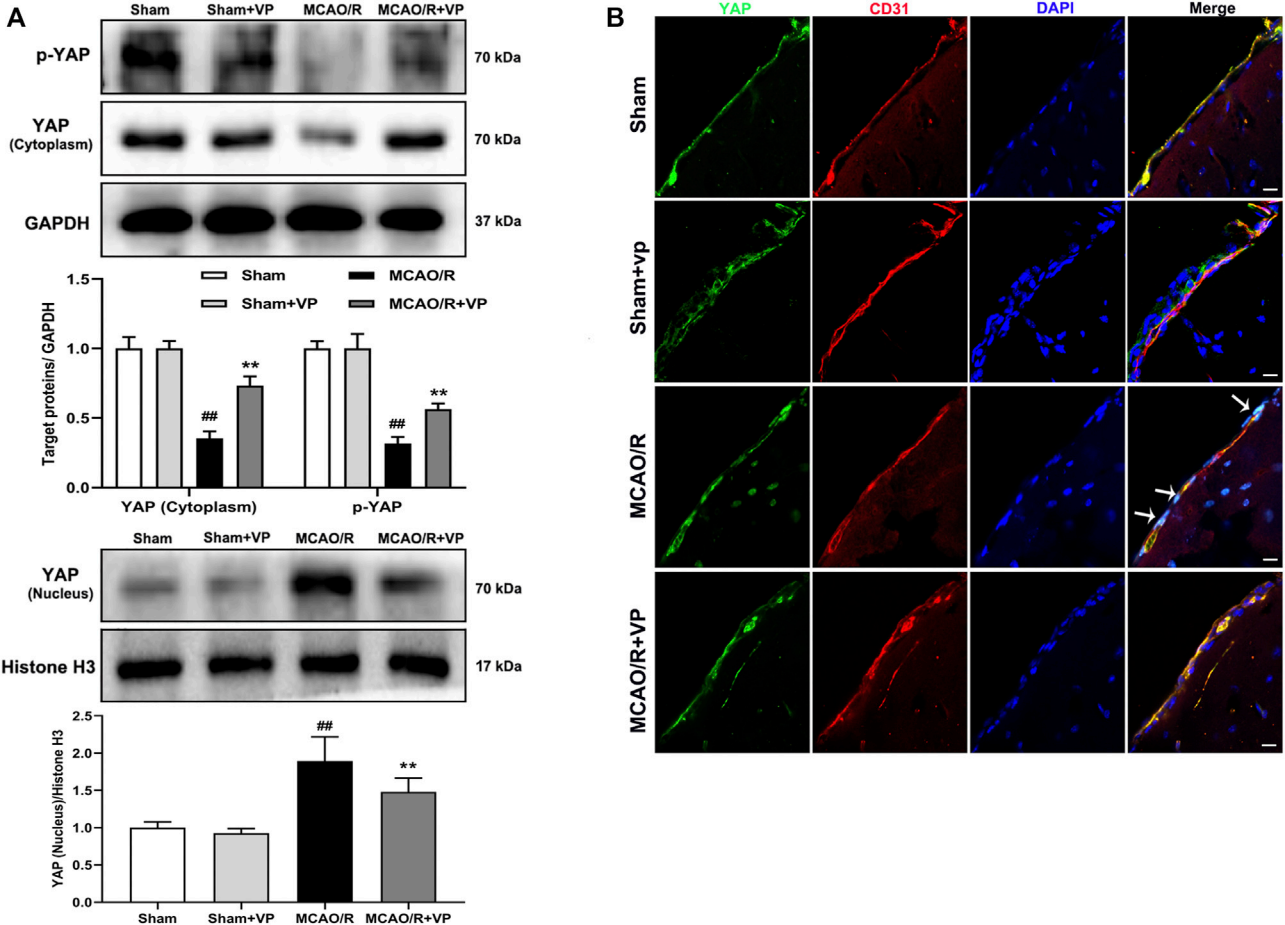
Effects of VP treatment on YAP/p-YAP expression levels following MCAO/R injury in mice. Mice were subjected to 1 h of ischemia and 24 h of reperfusion. **(A)** Representative western blots and quantitative analyses of the expression levels of p-YAP, YAP (cytoplasm), and YAP (nucleus) *in vivo*. **(B)** Representative microscopic images of YAP (green), based on immunofluorescence analyses. DAPI-stained nuclei are depicted in blue. The white arrow represents the number of YAP protein into the nucleus. Data are expressed as the mean ± SD, *n* = 6. ^##^ *p* < 0.01 *vs*. Sham group, **p < 0.01 *vs*. MCAO/R group. Scale bar = 50 µm.

The western blot analysis results showed that VP (10 mg/kg), when i. p. injected 1 h after MCAO, significantly increased the expression levels of p-YAP and YAP in the cytoplasm and decreased YAP expression levels in the nucleus (Figure 2A). In addition, the IF results also showed that the fluorescence intensity of p-YAP significantly increased (Supplementary Fig. 7), and the intensity of YAP in nucleus significantly decreased after the administration of VP compared with the intensities observed in the untreated MCAO/R group (Figure 2B and Supplementary Figure S6).

### 3.1 Inhibition of YAP Attenuates MCAO/R-Induced Brain Damage

**FIGURE 3:**
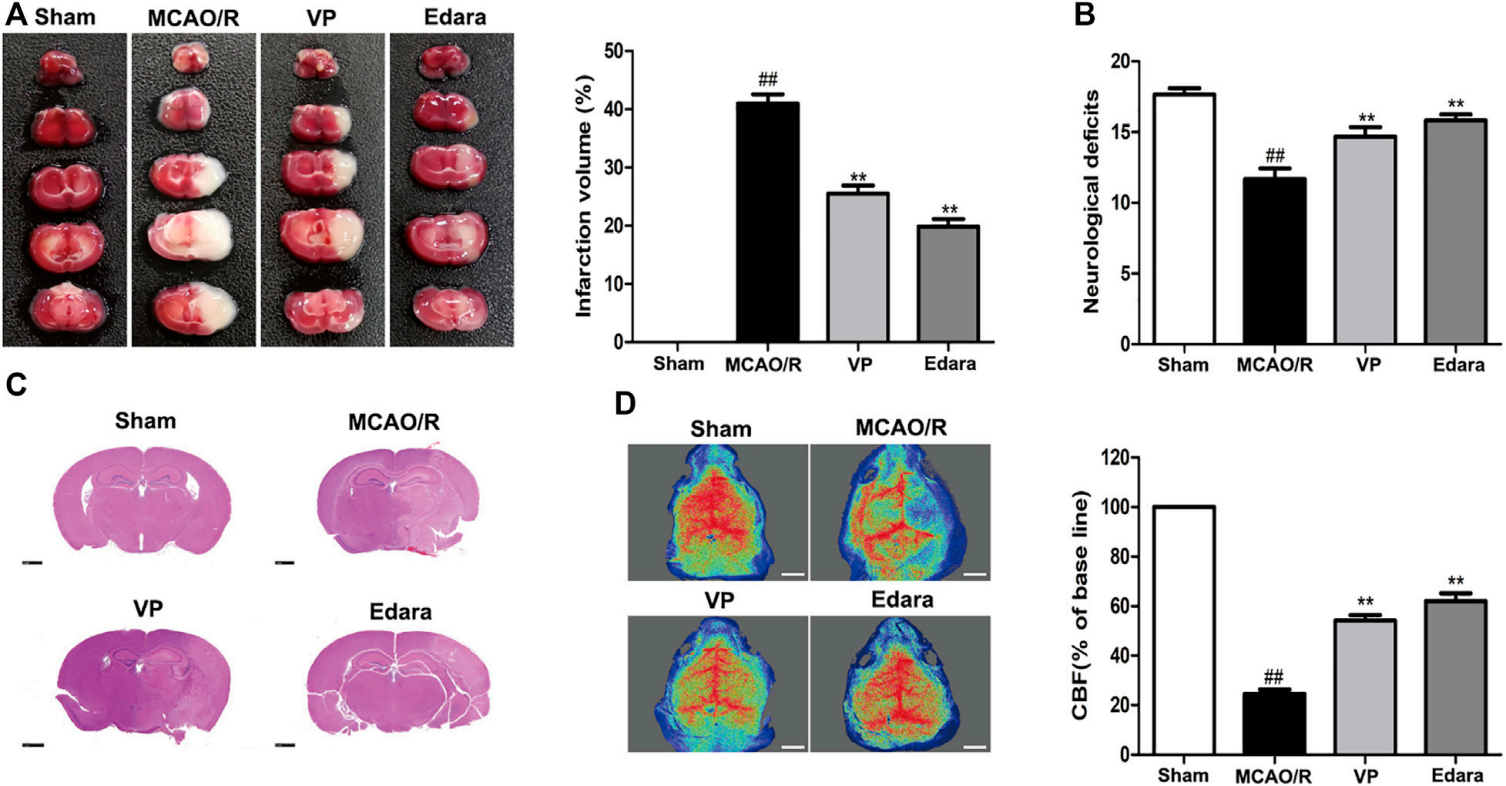
Effects of VP treatment on brain damage caused by MCAO/R injury in mice. Mice were subjected to 1 h of ischemia and 24 h of reperfusion. Mice were treated with VP (10 mg/kg) after 1 h of ischemia. **(A)** Mouse brains were evaluated for infarct volumes using TTC staining, which was quantified using imaging software, after I/R injury. **(B)** Quantitation of the neurological deficits measured in different groups. **(C)** H and E-stained images of mouse brain sections from different groups, examined under a light microscope. **(D)** CBF quantification in ischemic brain regions. Data are expressed as the mean ± SD, *n* = 6. ^##^ *p* < 0.01 *vs*. Sham group, **p < 0.01 *vs*. MCAO/R group. Scale bar = 50 µm.

VP (10 mg/kg) significantly reduced cerebral infarct volume, cerebral edema, and neurological deficits after MCAO/R injury (Figures 3A,B, Supplementary Figures S8 and 9). The damaged area of the brain was reduced significantly in MCAO/R model mice after the administration of VP compared with the untreated MCAO/R mice (Figure 3C). CBF improved in the ischemic hemisphere of MCAO/R mice after the administration of VP compared with untreated MCAO/R mice (Figure 3D). The efficacy of VP was similar to that observed for Edara, which was used as a positive control, which indicated that VP treatment induced improvements following ischemic brain injury in mice.

### 3.2 The Inhibition of YAP Results in the Maintenance of BBB Integrity Following MCAO/R Injury

**FIGURE 4:**
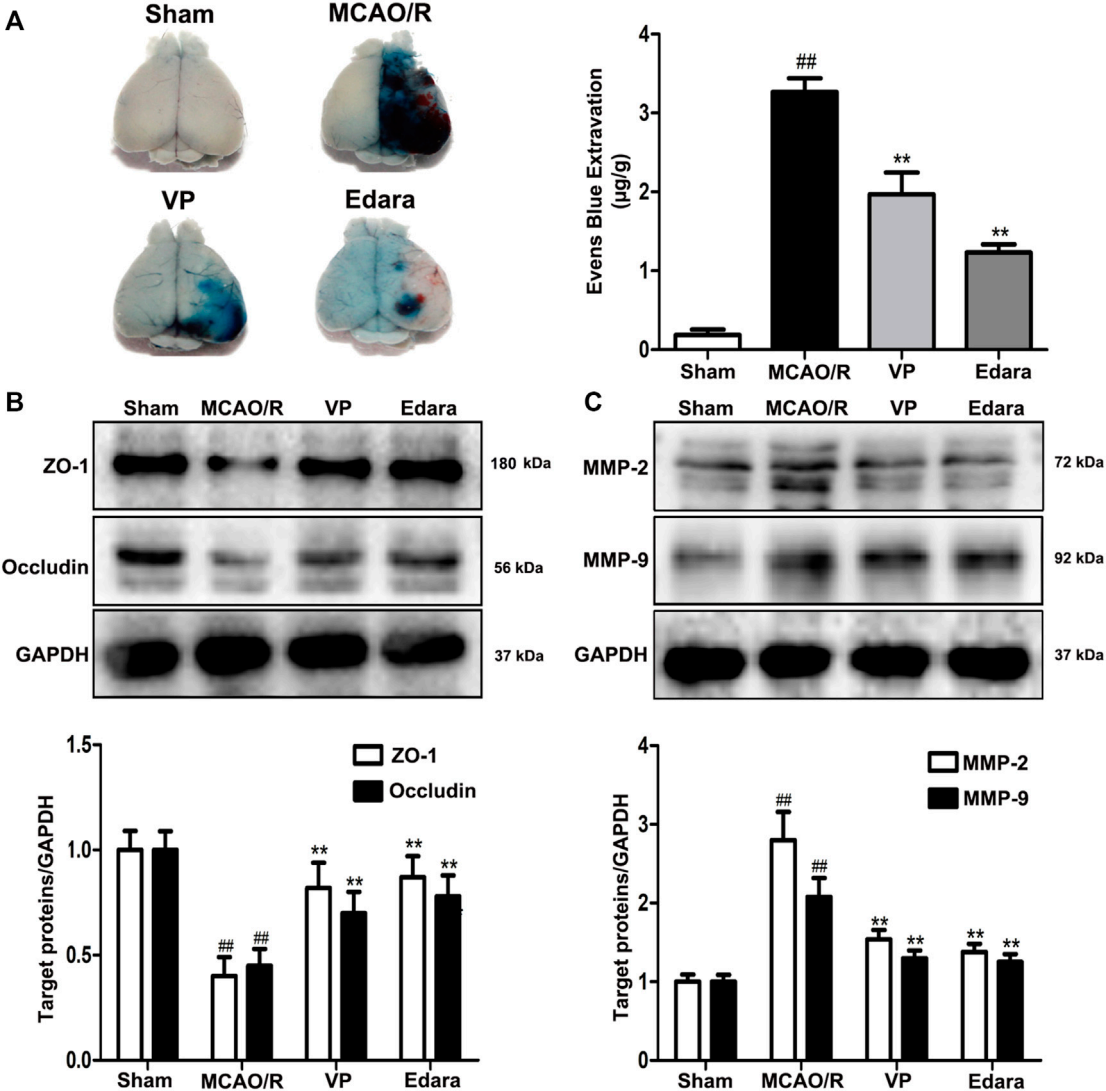
Effects of VP treatment on BBB damage caused by MCAO/R injury in mice. Mice were subjected to 1 h of ischemia and 24 h of reperfusion. Mice were treated with VP (10 mg/kg) after 1 h of ischemia. **(A)** Representative gross appearance of EB-stained brains from a mouse subjected to 1 h of ischemia followed by 24 h of reperfusion. **(B)** Representative western blots and quantitative analyses of ZO-1 and occludin expression levels after the administration of VP in mice. **(C)** Representative western blots and quantitative analyses of MMP-2/9 expression levels after the administration of VP in mice. Data are expressed as the mean ± SD, *n* = 6. ^##^ *p* < 0.01 *vs*. Sham group, **p < 0.01 *vs*. MCAO/R group.

Compared with the untreated MCAO/R group, the administration of VP significantly reduced EB leakage, increased the expression levels of ZO-1 and occludin, and decreased the expression levels of matrix metallopeptidase (MMP)-2 and MMP-9 in brain tissues (Figure 4). The efficacy of VP was similar to Edara, which indicated that VP could improve BBB integrity following MCAO/R injury.

### 3.3 YAP is Highly Expressed in the Nucleus of Cells Exposed to OGD/R Injury *In vitro*

**FIGURE 5:**
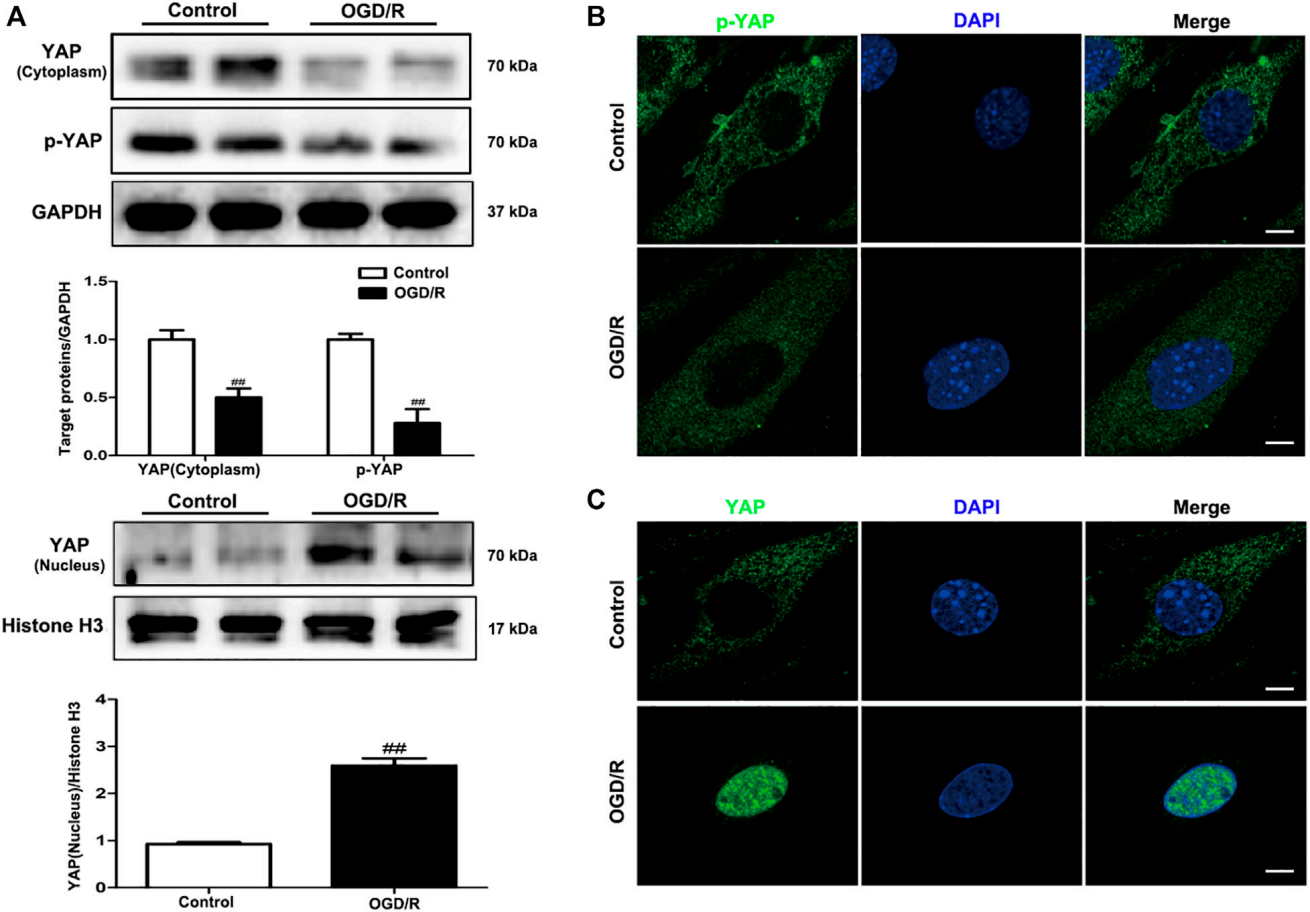
Expression levels of YAP/p-YAP in brain endothelial cells following OGD/R-induced injury. bEnd.3 cells were exposed to 6 h of OGD and 6 h of reoxygenation. **(A)** OGD/R-induced changes in YAP/p-YAP protein expression levels in the cytoplasm and nucleus, as detected by western blotting analysis. **(B)** and **(C)** OGD/R-induced changes in YAP/p-YAP protein expression levels were detected by immunofluorescence using a combination of anti-YAP (green), anti-p-YAP (green), and DAPI (blue) staining in bEnd.3 cells. Scale bar = 50 µm. Data are expressed as the mean ± SD, *n* = 3. ^##^ *p* < 0.01 *vs*. Control group.

To evaluate changes in YAP expression in an *in vitro* model of cerebral I/R injury, YAP/p-YAP expression levels were examined in the brain-derived EC line bEnd.3 following the induction of an OGD/R model. YAP/p-YAP expression levels in the cytoplasm reduced gradually after 6 h of OGD and 6 h of reoxygenation, whereas YAP expression increased gradually in the nucleus (Figure 5A). IF analysis also showed changes in YAP/p-YAP expression occurred after 6 h of OGD followed by 6 h of reoxygenation (Figure 5B), which was consistent with the results of *in vivo* studies.

### 3.4 VP Inhibits the Expression of YAP in the Nucleus Under OGD/R Injury Conditions

**FIGURE 6:**
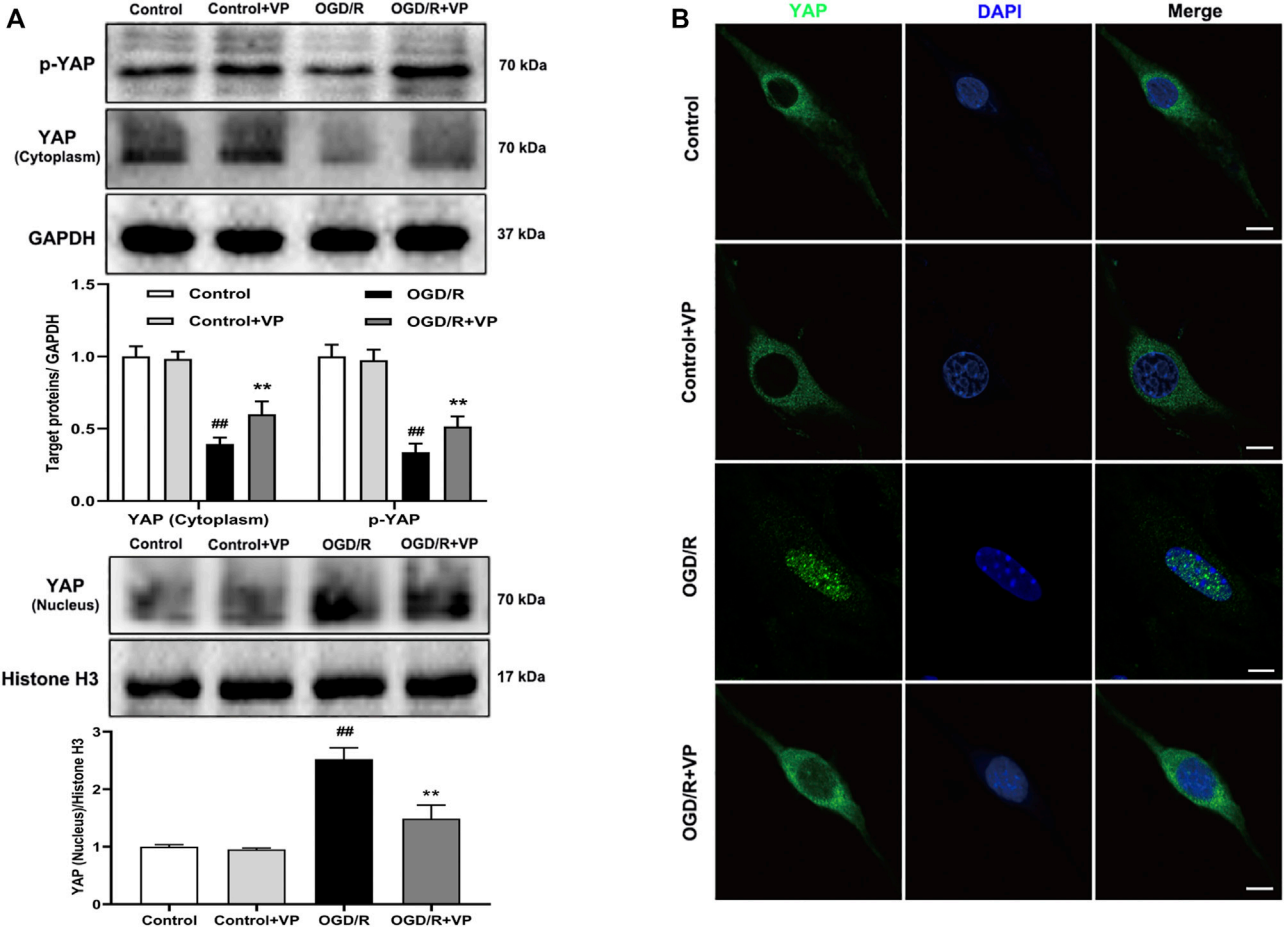
Effects of VP treatment on YAP/p-YAP expression levels following OGD/R injury in brain endothelial cells. bEnd.3 cells were treated with VP (1 µM) and subsequently exposed to 6 h of OGD and 6 h of reoxygenation. **(A)** Representative western blots and quantitative analyses showing the expression levels of p-YAP, YAP (cytoplasm), and YAP (nucleus) *in vitro*. **(B)** Representative microscopic images of YAP (green) based on immunofluorescence analyses. DAPI-stained nuclei are depicted in blue. Data are expressed as the mean ± SD, *n* = 6. ^##^ *p* < 0.01 *vs*. Control group, **p < 0.01 *vs*. OGD/R group. Scale bar = 50 µm.

Whether VP can inhibit the expression of YAP in the nucleus under OGD/R injury conditions remains to be elucidated. The expression levels of the Hippo pathway target kinase YAP and p-YAP in the Hippo pathway were measured by western blotting and IF analyses *in vitro* after the administration of VP. The results showed that VP (1 µM) significantly increased the expression levels of p-YAP and YAP in the cytoplasm and decreased YAP expression levels in the nucleus (Figures 6A–C). Further analysis showed that under OGD/R conditions, the ratio of translocated YAP protein into the nucleus was approximately 85%, and after VP administration, the ratio of translocated YAP into the nucleus was approximately 30% (Supplementary Figure S10).

### 3.5 Inhibition of YAP Ameliorates the Loss of Endothelial Barrier Integrity Induced by OGD/R Injury

**FIGURE 7:**
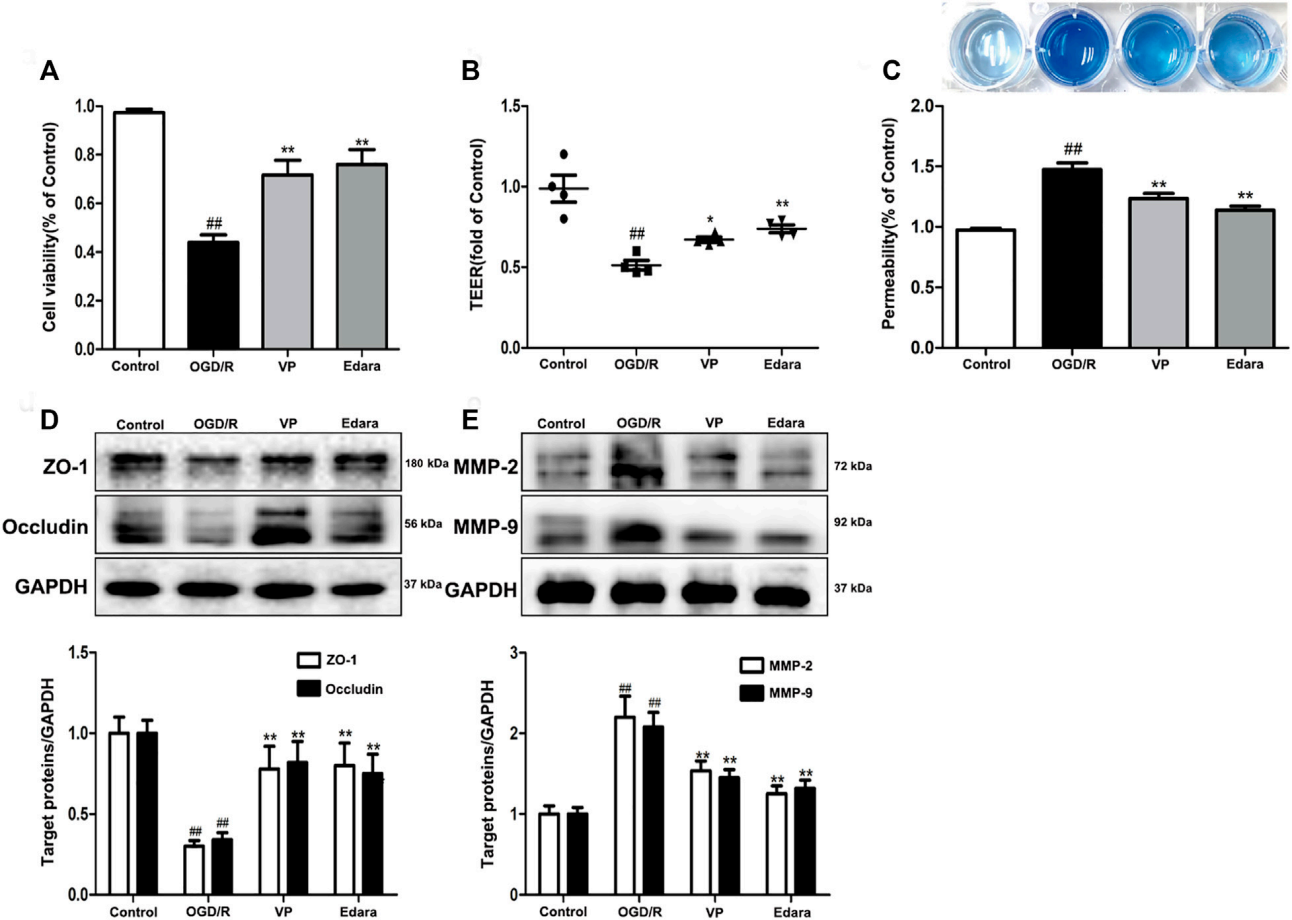
Effects of VP treatment on endothelial barrier injuries induced by OGD/R. bEnd.3 cells were treated with VP (1 µM) and subsequently exposed to 6 h of OGD and 6 h of reoxygenation. **(A)** The protective effect of VP in endothelial cells was detected by MTT assays. **(B)** and **(C)** The protective effect of VP on the endothelial cell barrier was detected using TEER and EB assays. **(D)** Representative western blots and quantitative analyses showing ZO-1 and occludin expression levels. **(E)** Representative western blots and quantitative analyses of MMP-2 and MMP-9 expression levels. Data are expressed as the mean ± SD, *n* = 3. ^##^ *p* < 0.01 *vs*. Control group, **p < 0.01 *vs*. OGD/R group.

To further investigate the protective effects of VP *in vitro*, bEnd.3 cells exposed to OGD/R injury were utilized. Compared with the control group, the cell viability of the OGD/R group decreased, based on the results of an MTT assay (Figure 7A, Supplementary Figure S11). Treatment with VP (1 µM) and Edara (1 µM) significantly increased cell survival following OGD/R injury. TEER was lower after OGD/R injury compared with the control group and increased significantly following treatment with VP and Edara (Figure 7B). EB leakage increased in the OGD/R group compared with that in the control group, and VP significantly inhibited OGD/R-induced EB leakage to an equivalent level as observed for Edara (Figure 7C). Compared with the OGD/R group, the protein expression levels of ZO-1 and occludin were increased, and MMP−2 and −9 expression levels were decreased significantly after VP administration (Figures 7D,E).

### 3.6 YAP-siRNA Attenuates OGD/R Injury-Induced Endothelial Barrier Disruption

**FIGURE 8:**
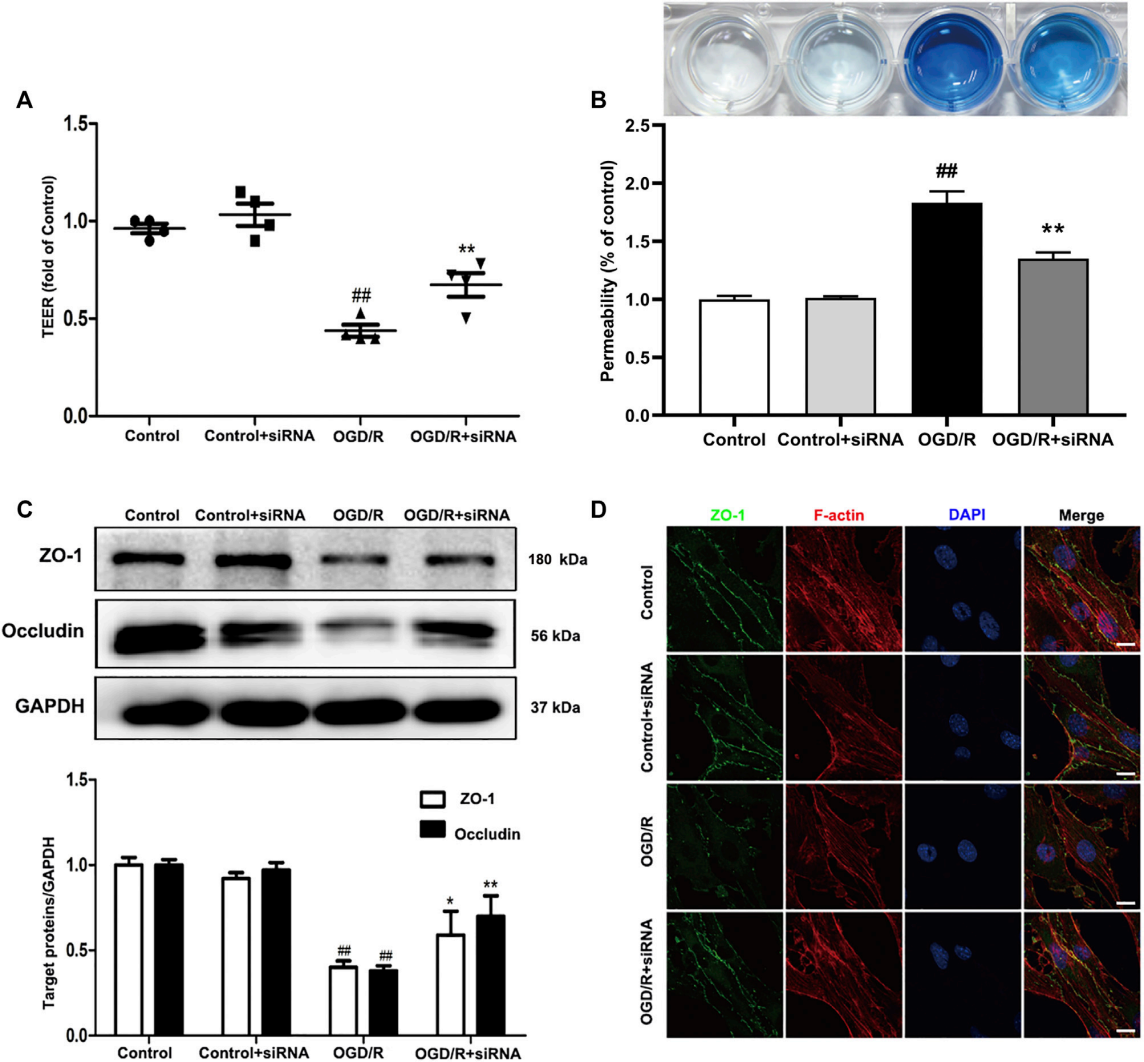
Effects of YAP-siRNA on endothelial barrier injury induced by OGD/R. bEnd.3 cells were treated with YAP-siRNA and subsequently exposed to 6 h of OGD and 6 h of reoxygenation. **(A)** and **(B)** A barrier-protective effect for YAP-siRNA was detected using TEER and EB assays. **(C)** Representative western blots and quantitative analyses for ZO-1 and occludin expression levels. **(D)** Representative microscope images (based on immunofluorescence analyses) of ZO-1 (green), F-actin (red), and DAPI-stained nuclei (blue). Scale bar = 20 µm. Data are expressed as the mean ± SD, *n* = 3. ^##^ *p* < 0.01 *vs*. Control group, *p < 0.05, **p < 0.01 *vs*. OGD/R group.

To further evaluate the effects of YAP on TJs between ECs, we knocked down YAP expression in bEnd.3 cells using YAP-siRNA *in vitro* (Supplementary Figures S11 and 12)*.* Compared with the control group, the TEER of cells subjected to OGD/R. Treatment with YAP-siRNA induced a significant increase in TEER (Figure 8A). EB leakage increased in the OGD/R group compared with the control group. YAP-siRNA significantly inhibited OGD/R-induced EB leakage to an equivalent level (Figure 8B). Compared with the OGD/R group, the protein expressions levels of ZO-1 and occludin increased significantly after the administration of YAP-siRNA (Figure 8C). The IF results also showed that the fluorescence intensity of ZO-1 increased significantly after the administration of YAP-siRNA compared with that in the OGD/R group (Figure 8D).

## 4 Discussion

Here, we identified a previously unrecognized role for YAP in the maintenance of endothelial TJ stability. The increased expression of YAP in the nucleus was observed in both cellular and animal models of cerebral I/R injury. The specific role played by YAP was investigated through the use of a YAP inhibitor and the use of YAP siRNA. The results showed YAP inhibition improved cerebral I/R injury-induced BBB dysfunction. We identified YAP as a regulator of BBB integrity during pathological injury. Thus, the inhibition of YAP expression during cerebral I/R injury may represent a novel strategy for the promotion of ischemic stroke recovery.

The Hippo/YAP signaling pathway plays an essential role in central nervous system development Bao et al. (2017). YAP participates in a range of cellular functions, including migration, adhesion, phagocytosis, and signal transduction Guichet et al. (2018). YAP regulates adherens junction dynamics and EC distribution during vascular development (Dieterich et al., 2000; Nagasawa-Masuda and Terai, 2017). The Hippo/YAP signaling pathway has been reported to be involved in the destruction of the BBB in an *in vivo* model (Gong et al., 2019; Jin et al., 2020). However, no studies have investigated the function of YAP in the maintenance of CEB integrity in mice and brain EC models of ischemic stroke. In this study, we found that YAP was highly expressed in the nucleus following cerebral I/R injury model induction (Figures 1–5), which indicated that the Hippo/YAP signaling pathway was linked to CEB injury after ischemic stroke. We postulate that YAP acts as a key target protein, which participates in the pathological process and biologic function of ischemic stroke; however, few studies have investigated the involvement of YAP in CEB regulation in ischemic stroke.

To further explore the role played by YAP during brain injury induced by cerebral I/R, we used a YAP inhibitor, verteporfin (VP), which is a benzoporphyrin derivative that is clinically used in photodynamic therapy for neovascular macular degeneration (Brodowska et al., 2014). Recently, studies have shown that VP inhibits YAP activation by disrupting YAP-TEA domain transcription factor (TEAD) interactions, which prevented YAP-induced oncogenic growth (Liu-Chittenden et al., 2012). However, whether and how VP regulates YAP expression during the development of ischemic stroke remains unknown. In this study, VP treatment was found to significantly increase the expression levels of p-YAP and YAP in the cytoplasm and decreased YAP expression levels in the nucleus under cerebral I/R model conditions (Figures 2–6). Furthermore, VP treatment was able to reduce the cerebral infarct volume and brain water contents and improve neurological deficits and CBF in cerebral I/R model mice. Cerebral infarct volume, neurological deficits, brain edema, and CBF are often used to evaluate the degree of brain injury (Lochhead et al., 2017). H and E staining is an important method used to evaluate the degree of pathological changes in tissue sections (Campbell et al., 2017). Our results suggested that VP ameliorated MCAO/R-induced brain damage *in vivo* (Figure 3, Supplemental Figure S8).

The evolution of BBB breakdown after cerebral I/R occurs along the following path: I/R rapidly induces cytoskeletal alterations in BMECs, due to the activation of a variety of protease and signaling pathways. Cytoskeletal alterations cause EC contraction and the disassembly of TJs through junctional-accessory proteins (for example, ZO-1, occludin). The disassembly and redistribution of TJs result in subtle BBB hyperpermeability, inducing the extravasation of fluid and small macromolecules from the blood to the central nervous system. The weakened barrier becomes more vulnerable to the MMP-2/9-mediated degradation of TJs, further damaging the BBB and permitting the eventual leakage of large macromolecules Shi et al. (2016). *In vivo*, EB staining is often used to evaluate the degree of BBB damage (Nishiyama et al., 2008). VP treatment in MCAO/R model mice was able to significantly decrease EB leakage and MMP2/9 expression levels and increase the expression levels of ZO-1 and occludin compared with untreated MCAO/R model mice (Figure 4). *In vitro*, VP remarkably alleviated OGD/R-induced endothelial-barrier injury, mitigated bEnd.3 cell leakage, and inhibited the degradation of TJ proteins (Figure 7) in OGD/R exposed cells compared with the untreated control, indicating the protective effects of VP against I/R-induced CEB damage. Meanwhile, bEnd.3 cells transfected with YAP-siRNA were used to evaluate the effects of YAP on endothelial barrier integrity *in vitro*. YAP expression decreased in following siRNA interference in bEnd3 cells exposed to OGD/R (Figure 8). These results indicated that YAP-siRNA was able to maintain the integrity of the endothelial barrier by promoting the preservation of TJ, which further indicated that YAP is a vital target molecule for the maintenance of BBB integrity.

Cerebral ECs are key components involved in the maintenance of BBB integrity. The loss of BBB integrity is a pathophysiological hallmark of brain diseases, including Alzheimer’s disease, epilepsy, and cranial trauma (Straight et al., 2003; Schmidt et al., 2014; Sharma and Goyal, 2016). Studies have reported that Hippo (MST)-YAP signaling is involved in brain vessel in various diseases, including cerebral I/R injury and subarachnoid hemorrhage, suggesting the potential for the modulation of this signaling pathway to influence the prognosis of many types of neurological disorders (Gong et al., 2019; Zhao et al., 2016; Qu et al., 2018). Future studies remain necessary to elucidate the specific roles played by this pathway in the development of these various neurological disorders. As an essential component of this signaling pathway, YAP has been shown to be involved in several diseases, including the discord caused by viruses or bacteria (Lee et al., 2013). Our results suggested that YAP might act as a key mediator in I/R-induced CEB injury. Our findings provide broad insights into brain injury characterized by BBB hyperpermeability and indicate new therapeutic strategies for severe diseases associated with dysfunctional TJ signaling.

**FIGURE 9:**
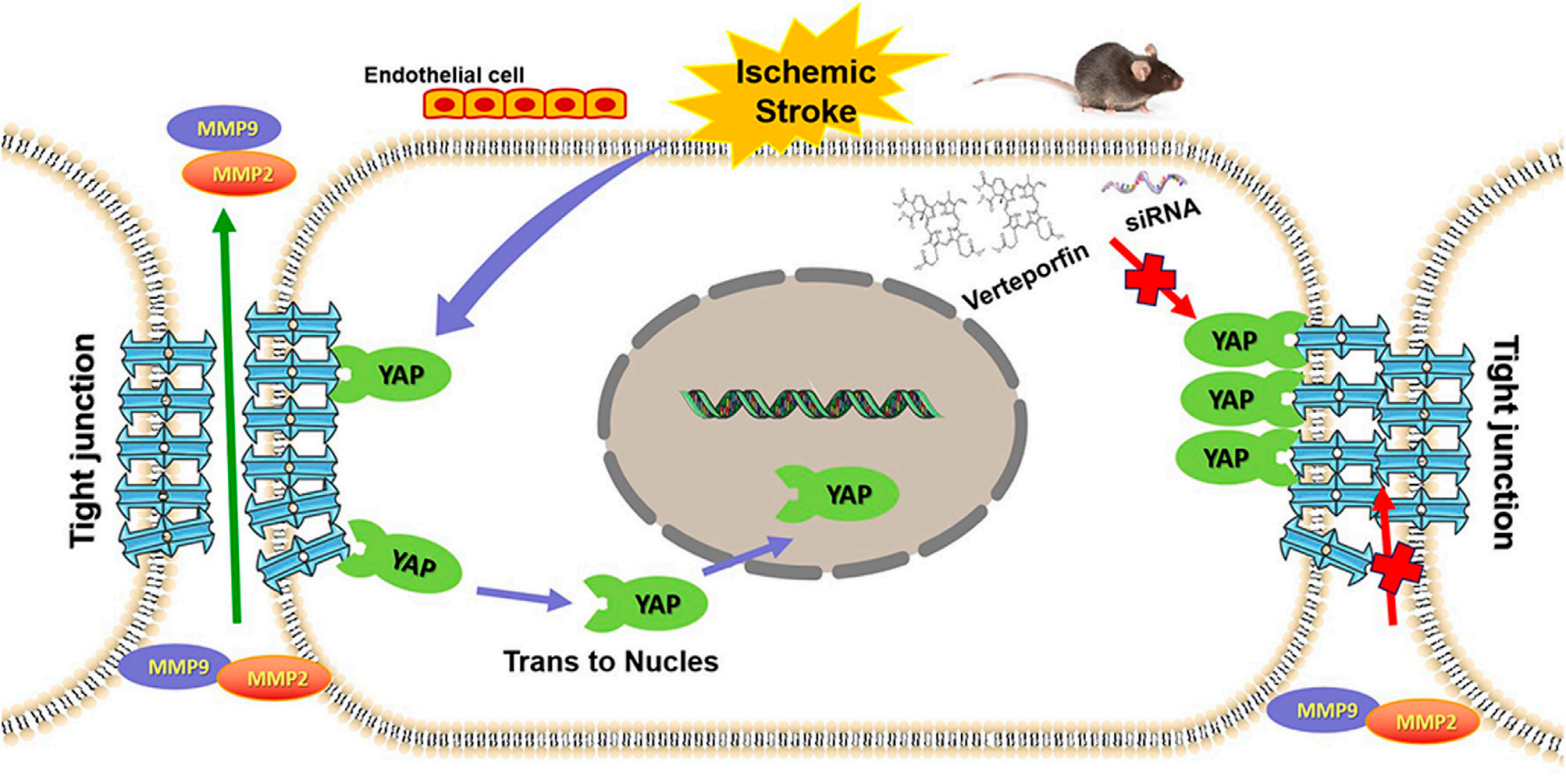
Graphic abstract depicting the role played by YAP to protect against BBB disruption in cerebral ischemia-reperfusion injury.

In summary, the inhibition of YAP expression in the nucleus beneficially antagonizes the high endothelial permeability induced by cerebral I/R injury, both *in vivo* and *in vitro*. As a regulatory molecule, YAP contributes to the maintenance of CEB integrity (Figure 9). Taken together, our findings extend the current understanding of the regulatory mechanisms associated with TJ function and present potential novel targets for the development of efficacious drugs that may prevent and treat damage associated with ischemic stroke and other related diseases.

In conclusion, we clarified a key role for YAP in BBB maintenance during stroke. YAP could represent a potential target in ECs for pharmacotherapeutic interventions designed to protect the BBB. Our data revealed new opportunities for the prevention of brain damage aggravation following ischemic stroke.

## Data Availability

The original contributions presented in the study are included in the article/Supplementary Material, further inquiries can be directed to the corresponding authors.
