# Supplementary material for: Inhibiting YAP in Endothelial Cells From Entering the Nucleus Attenuates Blood-Brain Barrier Damage During Ischemia-Reperfusion Injury
Source: Front Pharmacol. 2021 Nov 26;12:777680. doi: 10.3389/fphar.2021.777680 (PMC8662521; doi:10.3389/fphar.2021.777680)

**Inhibiting YAP in endothelial cells from entering the nucleus attenuates blood-brain barrier damage during ischemia-reperfusion injury**


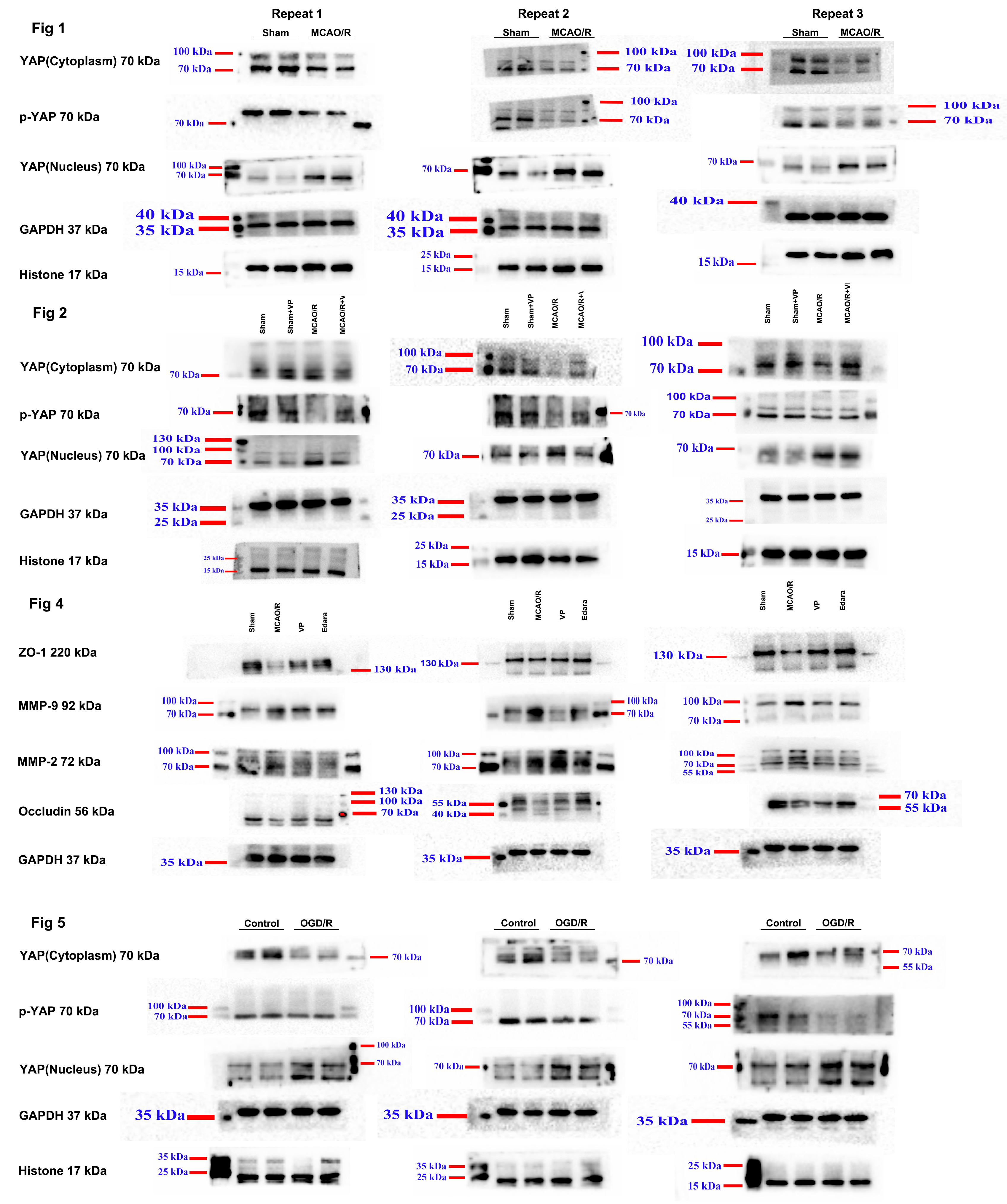

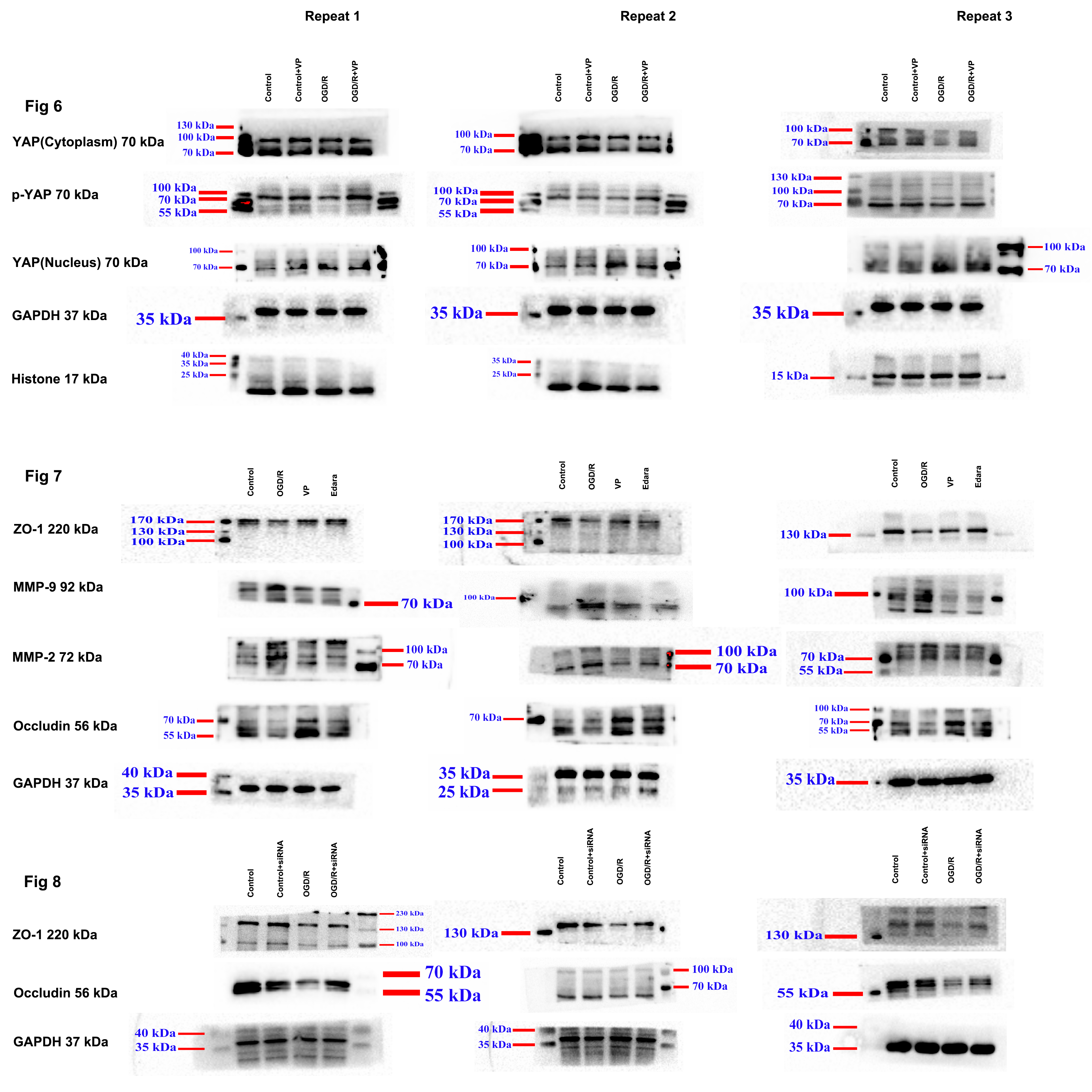

Supplement: Supplementary file 1 [file DataSheet2.doc]
