# Supplementary material for: Inhibiting YAP in Endothelial Cells From Entering the Nucleus Attenuates Blood-Brain Barrier Damage During Ischemia-Reperfusion Injury
Source: Front Pharmacol. 2021 Nov 26;12:777680. doi: 10.3389/fphar.2021.777680 (PMC8662521; doi:10.3389/fphar.2021.777680)
Supplement: Supplementary file 2 [file DataSheet1.docx]

**Supplementary Materials**

**
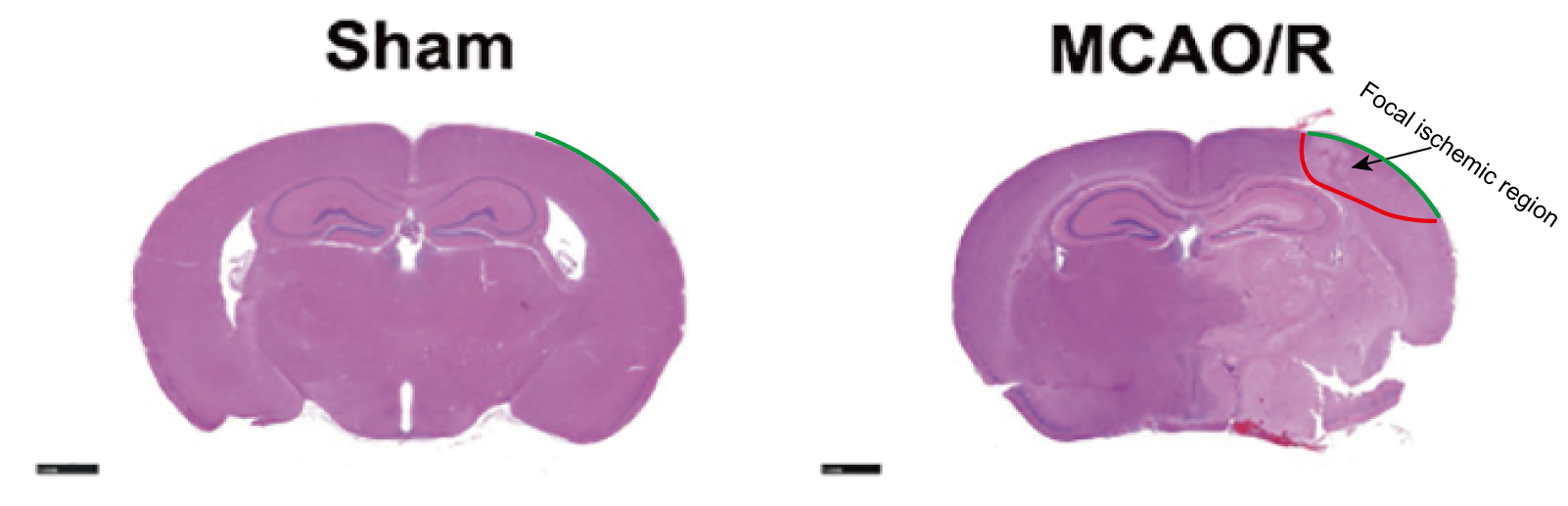
**

**Supplementary Fig. 1** The illustration figure on the location of studied brain area for the IF brain slice. The green part was selected for *vivo* immunofluorescence.

, **
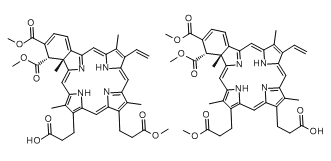
**

**Supplementary Fig. 2** Chemical structure of verteporfin (VP).


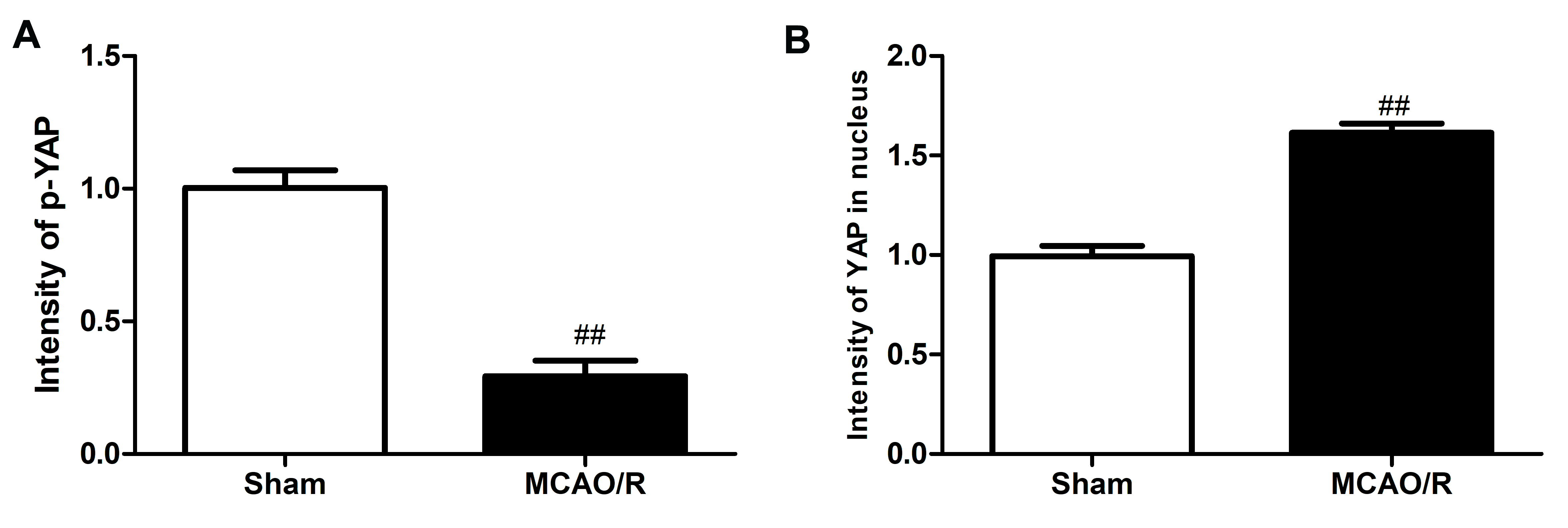


**Supplementary Fig. 3 Expression of YAP/p-YAP in brain injury induced by MCAO/R.** Mice were subjected to 1 h of ischemia and 24 h of reperfusion. MCAO/R-induced YAP/p-YAP proteins expression was detected by immunofluorescence using a combination of anti-YAP (green), anti-p-YAP (green), CD31(red) and DAPI (blue) staining in mice. Scale bar=50 µm. Data are the mean ± SD, n=3. ^##^*P*<0.01 *vs*. Sham group.

***
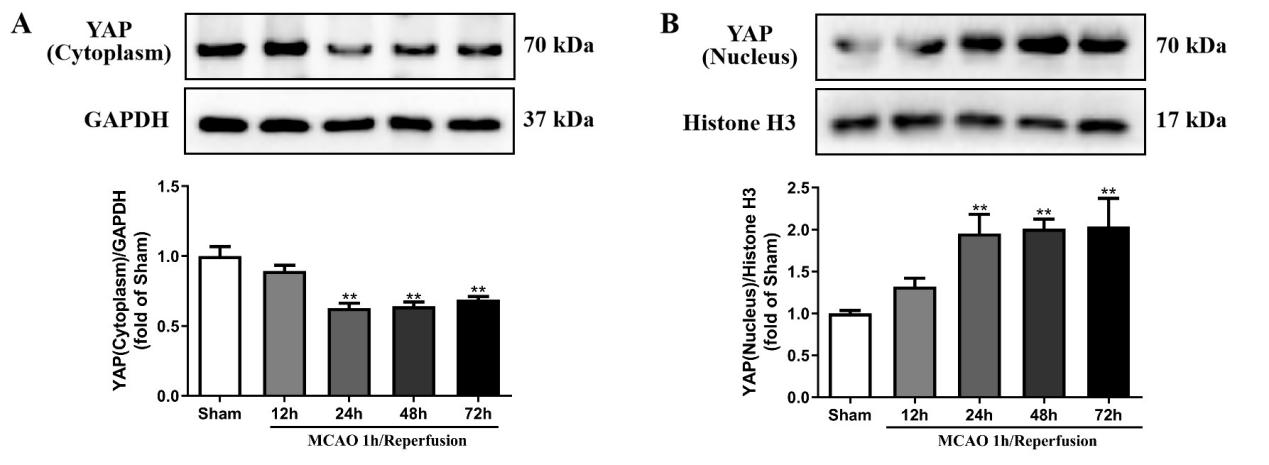
***

**Supplementary Fig. 4** **Change of YAP proteins during the progress of MCAO/R.** (A) Representative western blot of YAP (Cytoplasm) protein. (B) Representative western blot of YAP (Nucleus) protein. Quantitative analyses of expression of YAP. Data are shown as Mean±SEM, n=3. ***P*<0.01 vs. Sham group.

***
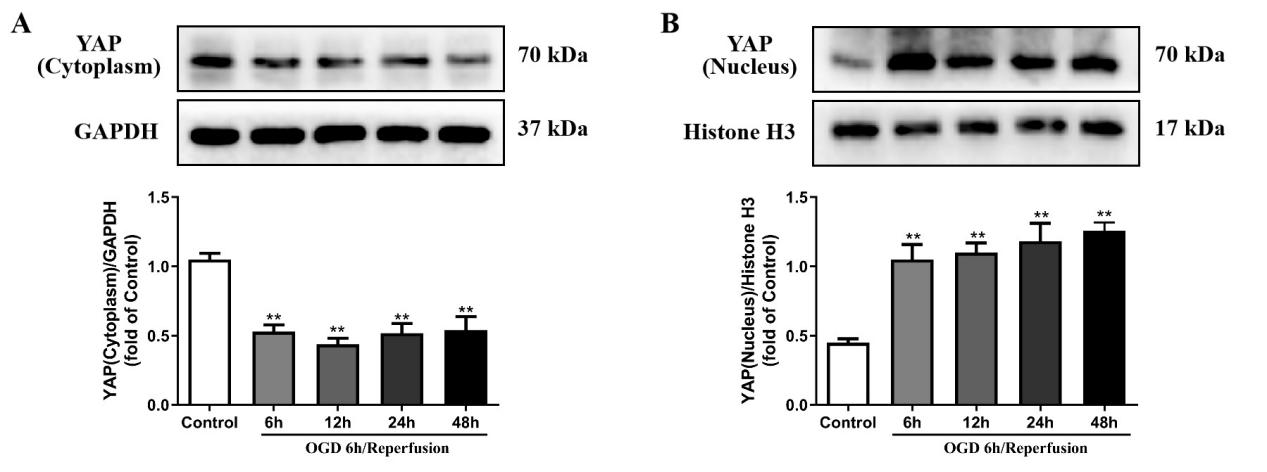
***

**Supplementary Fig. 5** **Change of YAP proteins during the progress of OGD/R.** (A) Representative western blot of YAP (Cytoplasm) protein. (B) Representative western blot of YAP (Nucleus) protein. Quantitative analyses of expression of YAP. Data are shown as Mean±SEM, n=3. ***P*<0.01 vs. Control group.


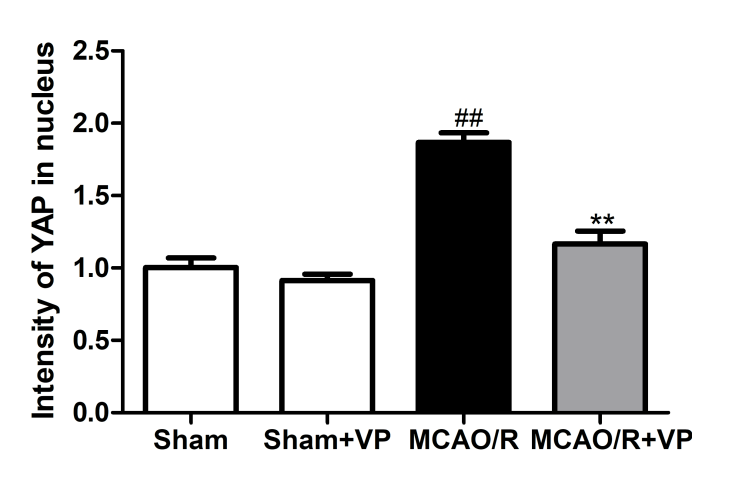


**Supplementary Fig. 6 Effect of VP on the intensity of YAP in nucleus caused by MCAO/R injury in mice.** Mice were subjected to 1 h of ischemia and 24 h of reperfusion. Representative microscope images (based on immunofluorescence analyses) of p-YAP (green), CD31 (red), and DAPI-stained nuclei are depicted in blue. Scale bar=50 µm. Data are the mean ± SD, n=3. ^##^*P*<0.01, *vs*. Sham group, ***P*<0.01, *vs*. MCAO/R group.


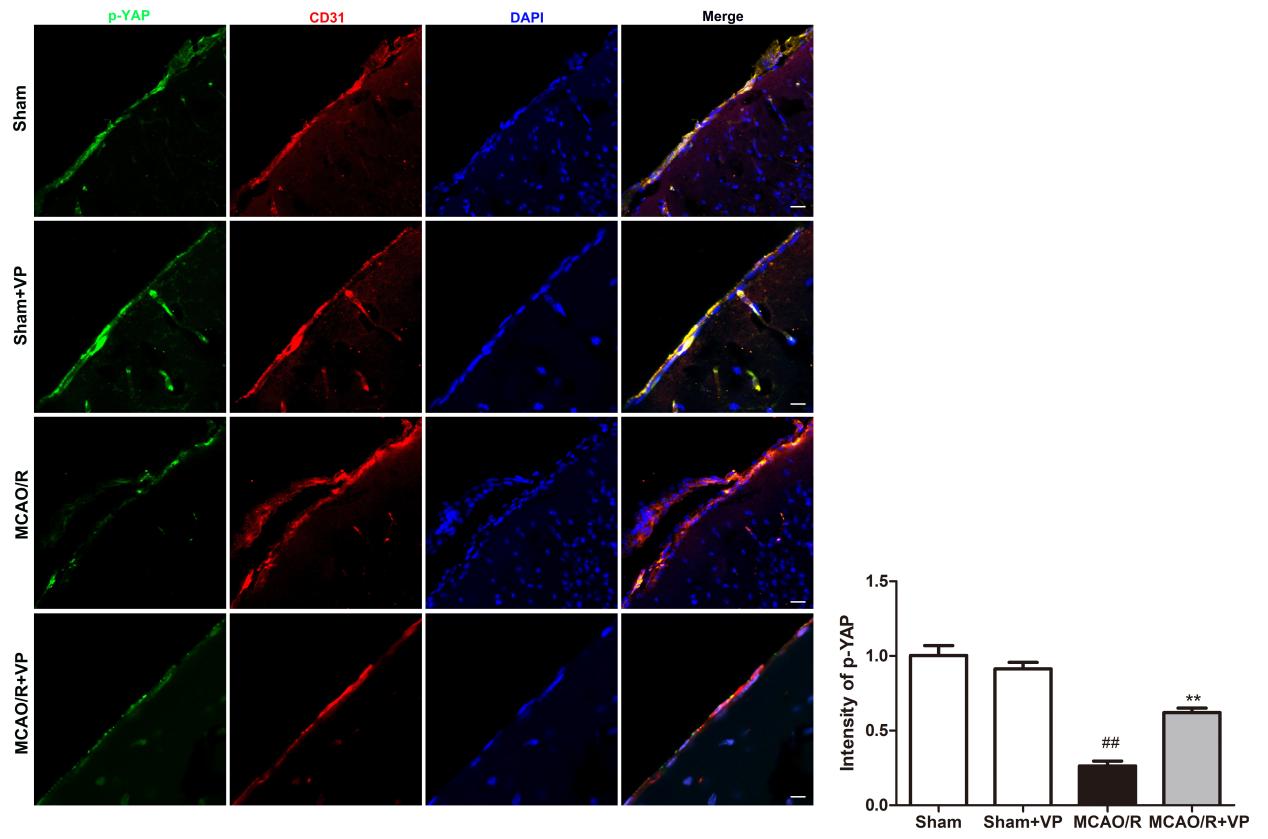


**Supplementary Fig. 7 Effect of VP on the intensity of p-YAP caused by MCAO/R injury in mice.** Mice were subjected to 1 h of ischemia and 24 h of reperfusion. Representative microscope images (based on immunofluorescence analyses) of p-YAP (green), CD31 (red), and DAPI-stained nuclei are depicted in blue. Data are the mean ± SD, n=3. ^##^*P*<0.01, *vs*. Sham group, ***P*<0.01, *vs*. MCAO/R group. Scale bar=50 µm.

**
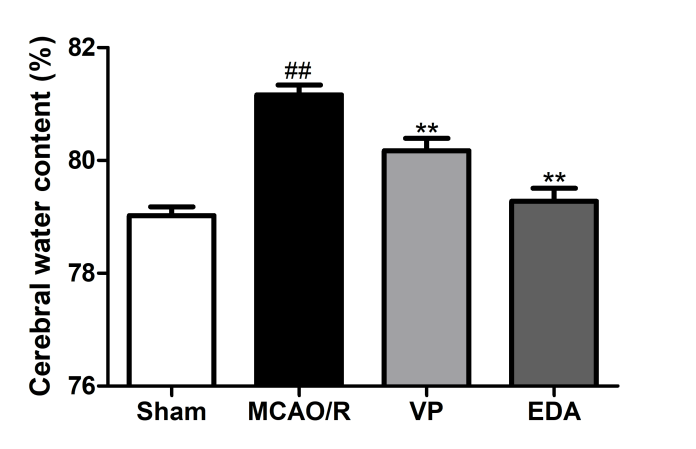
**

**Supplementary Fig. 8** **Effects of VP on the degree of cerebral edema caused by MCAO/R in mice.** Mice were subjected to 1 h of ischemia and 24 h of reperfusion. Mice were treated with VP (10 mg/kg) after 1 h of ischemia. Data are the mean ± SD, n=6. ^##^*P*＜0.01, *vs*. Sham group, ***P*<0.01, *vs*. MCAO/R group.


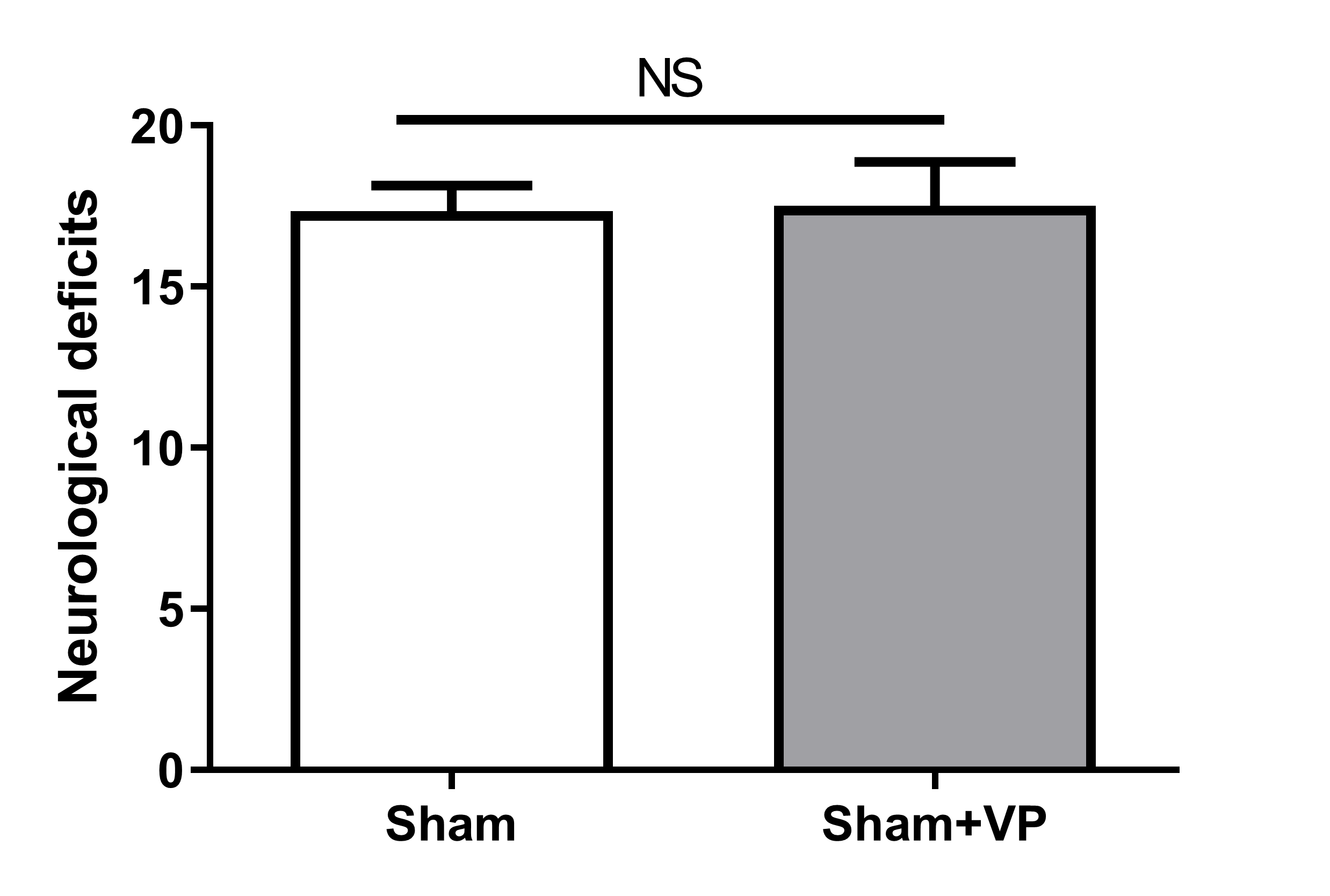


**Supplementary Fig. 9 Effects of VP on the degree of neurological deficits in sham group.** Quantitation of the neurological deficits measured in different groups. Data are expressed as the mean ± SD, n = 6.


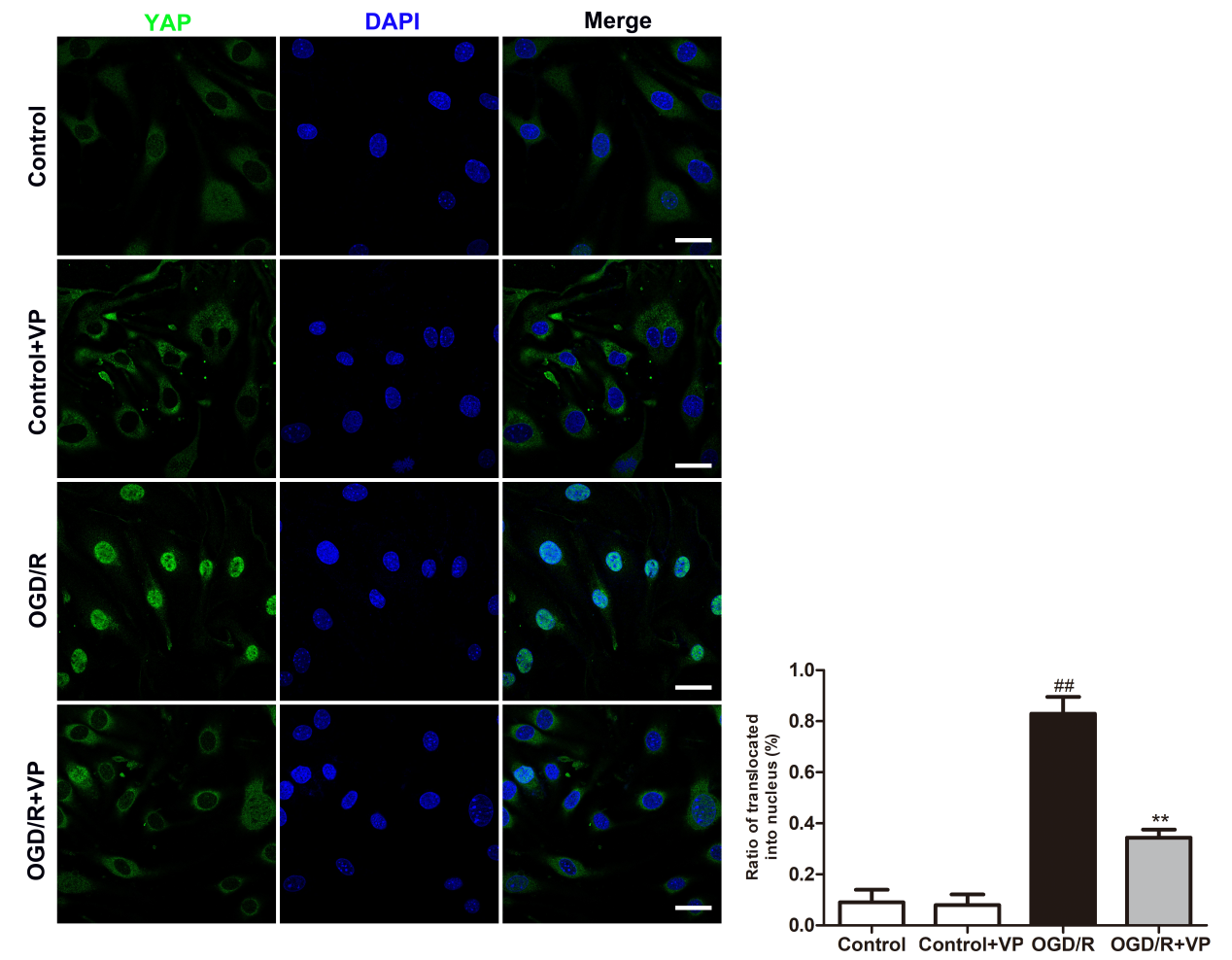


**Supplementary Fig. 10 Effect of VP on the ratio of translocated YAP into nucleus caused by OGD/R injury in brain endothelial cells.** bEnd.3 cells were treated with VP (1 µM) and exposed subsequently to 6 h of OGD and 6 h of reoxygenation. Representative microscope images of YAP (green) based on immunofluorescence analyses. DAPI-stained nuclei are depicted in blue. Data are the mean ± SD, n=6. ^##^*P*<0.01, *vs*. Control group, ***P*<0.01, *vs*. OGD/R group. Scale bar=20 µm.

**
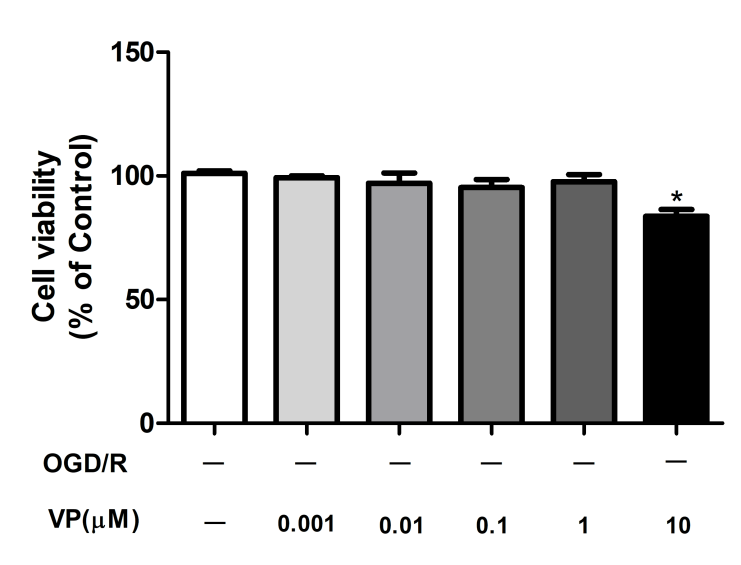
**

**Supplementary Fig. 11** **The effect of VP was detected using MTT assays in endothelial cell.** bEnd.3 cells were treated with VP and exposed subsequently to 6 h of OGD and 6 h of reoxygenation. Data are the mean ± SD, n=3. ^##^*P*<0.01 *vs*. Control group.

**
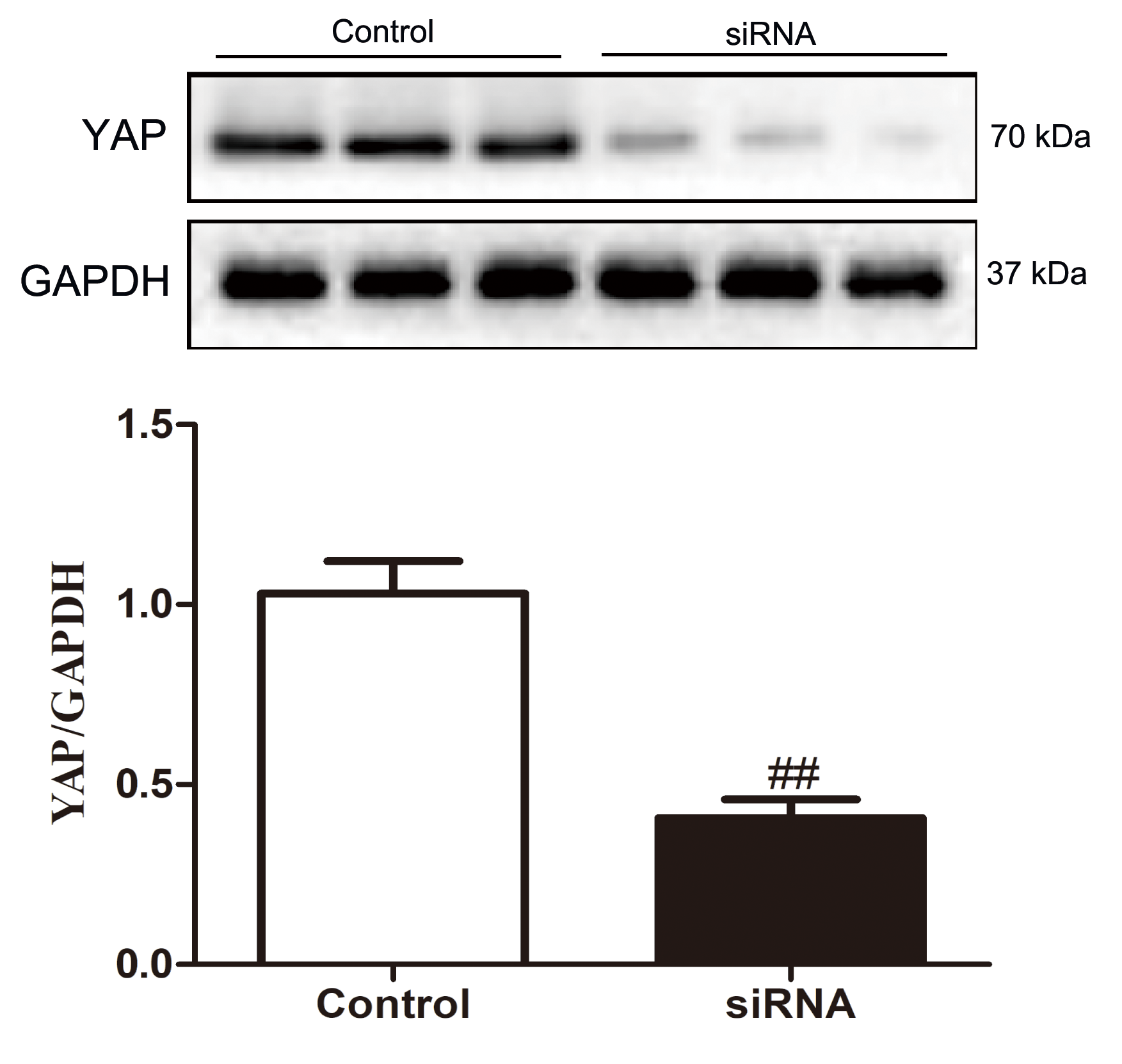
**

**Supplementary Fig. 12** **Screening for siRNA sequences.** Representative western blots and quantitative analyses of expression of YAP. Data are the mean ± SD, n=3. ^##^*P*<0.01 *vs*. Control group.


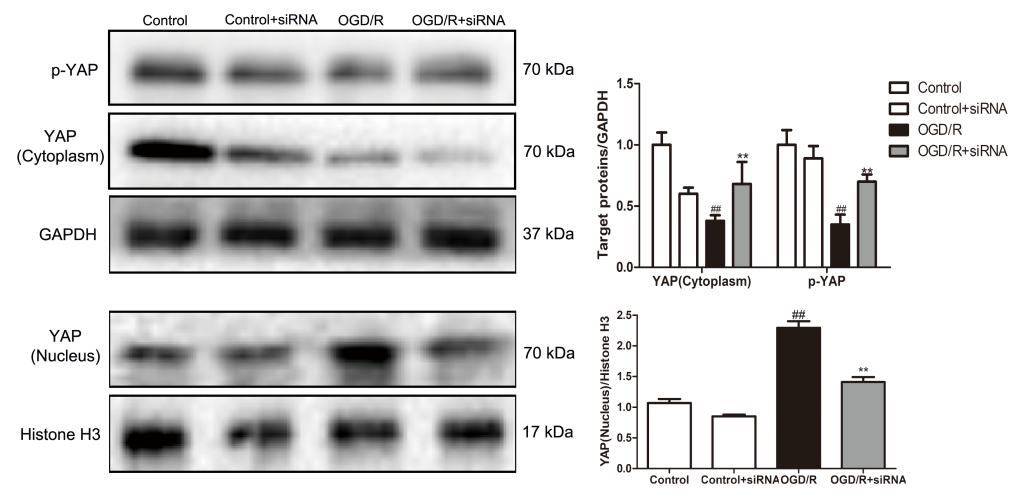


**Supplementary Fig. 13** **Effect of YAP siRNA on expression of YAP/p-YAP proteins in bEnd.3 cells.** Representative western blots and quantitative analyses of expression of p-YAP, YAP (cytoplasm), YAP (nucleus) . Data are the mean ± SD, n=3. ^##^*P*<0.01 *vs*. Control group, ***P*<0.01 *vs*. OGD/R group.
